# Enhancement of adsorption efficiency of crystal violet and chlorpyrifos onto pectin hydrogel@Fe_3_O_4_-bentonite as a versatile nanoadsorbent

**DOI:** 10.1038/s41598-023-38005-z

**Published:** 2023-07-04

**Authors:** Paria Beigi, Fatemeh Ganjali, Fereshte Hassanzadeh-Afruzi, Mohammad Mehdi Salehi, Ali Maleki

**Affiliations:** 1 Department of Physics, Iran University of Science and Technology, Tehran, 16846–13114 Iran; 2 Catalysts and Organic Synthesis Research Laboratory, Department of Chemistry, Iran University of Science and Technology, Tehran, 16846-13114 Iran

**Keywords:** Environmental sciences, Chemistry, Materials science, Nanoscience and technology

## Abstract

The magnetic mesoporous hydrogel-based nanoadsornet was prepared by adding the ex situ prepared Fe_3_O_4_ magnetic nanoparticles (MNPs) and bentonite clay into the three-dimentional (3D) cross-linked pectin hydrogel substrate for the adsorption of organophosphorus chlorpyrifos (CPF) pesticide and crystal violet (CV) organic dye. Different analytical methods were utilized to confirm the structural features. Based on the obtained data, the zeta potential of the nanoadsorbent in deionized water with a pH of 7 was − 34.1 mV, and the surface area was measured to be 68.90 m^2^/g. The prepared hydrogel nanoadsorbent novelty owes to possessing a reactive functional group containing a heteroatom, a porous and cross-linked structure that aids convenient contaminants molecules diffusion and interactions between the nanoadsorbent and contaminants, viz., CPF and CV. The main driving forces in the adsorption by the Pectin hydrogel@Fe_3_O_4_-bentonite adsorbent are electrostatic and hydrogen-bond interactions, which resulted in a great adsorption capacity. To determine optimum adsorption conditions, effective factors on the adsorption capacity of the CV and CPF, including solution pH, adsorbent dosage, contact time, and initial concentration of pollutants, have been experimentally investigated. Thus, in optimum conditions, i.e., contact time (20 and 15 min), pH 7 and 8, adsorbent dosage (0.005 g), initial concentration (50 mg/L), T (298 K) for CPF and CV, respectively, the CPF and CV adsorption capacity were 833.333 mg/g and 909.091 mg/g. The prepared pectin hydrogel@Fe_3_O_4_-bentonite magnetic nanoadsorbent presented high porosity, enhanced surface area, and numerous reactive sites and was prepared using inexpensive and available materials. Moreover, the Freundlich isotherm has described the adsorption procedure, and the pseudo-second-order model explained the adsorption kinetics. The prepared novel nanoadsorbent was magnetically isolated and reused for three successive adsorption–desorption runs without a specific reduction in the adsorption efficiency. Therefore, the pectin hydrogel@Fe_3_O_4_-bentonite magnetic nanoadsorbent is a promising adsorption system for eliminating organophosphorus pesticides and organic dyes due to its remarkable adsorption capacity amounts.

## Introduction

Along with the rapid population increase in the world, supplying food is a significant issue that has to be considered profoundly. In this regard, it seems inevitable not to use plant pests for agriculture in order to supply food. However, this procedure releases pesticides in nature and their excessive utilization^1,2^. Organophosphorus pesticides are categorized as synthetic pesticides among various pesticide types, which have been employed in industrial agriculture and utilized as insecticides as well as nerve agents in many countries because of the widespread restrictions on organochloride pesticide usage since the 1970s. Near 36% of the total world market of pesticides is consisted of organophosphorus ones^3^. Chlorpyrifos (CPF) is a chlorinated organophosphate insecticide with high crystallinity, which has been produced and utilized worldwide since 1965 in various forms, i.e., liquid, gel, pellets, wettable powders, etc.^4,5^. The public’s desire to utilize the CPF pesticide is related to two main reasons, one is its inexpensiveness and the other is its facile access^6^. The long-lasting durability of the CPF is assigned to its physicochemical features and structural characteristics. CPF is a nonpolar and water low-soluble material with increased partition from aquatic solvents to organic solvents. Because of suppressing the acetylcholinesterase enzyme, similar to other organophosphate forms, the CPF toxic effects occur, which could cause various neurobehavioral effects^7,8^. Due to a novel statement, researchers have considered diverse CPF effects on target cells.

Moreover, exposure to small amounts of this pesticide causes genetic disorders in humans and genotoxicity^9^. Organic dyes are toxic, low-biodegradable materials that have carcinogenic impacts on the aquatic organisms according to their stable and aromatic structures^10–12^. One of the widely utilized organic dye with positively-charged nature in industrial applications, including textile, pharmaceutics, etc. is crystal violet (CV)^13^. Releasing CV in the water sources has become a massive issue because of its threat for mammalian cells for its mutagenic poisonous effects that cause cancer, skin irritation, and cornea complications^14^. In spite of CPF and CV advantages in industrial applications, namely agriculture and textile, these substances cause severe problems by polluting the environment and threaten human health. Due to the abovementioned downsides, the treatment of the contaminated water from these organic pollutants seems crucial to protect the environment and human health. Until date, various physicochemical procedures have been applied for organic contaminants’ elimination, such as precipitation^15^, photocatalysis^16^, adsorption^17–19^, membrane filtration^20^, degradation^21^, ion exchange^22^, etc. Although the stated procedures render highlighted profits, they have several drawbacks in large-scale performance. For instance, forming a concentrated sludge is a time and energy consuming process with low efficacy and expensive operation. Therefore, among all of the processes for contaminant removal from water, adsorption is suggested due to its economical and practicable procedure, enhanced efficacy, selectivity and retrievability, and affordability^12,23^.

In this line, many adsorption systems have been designed and employed for contaminant elimination such as porous substances^24^, bioadsorbents^25^, carbon nanotubes^26^, graphene-based composites^27^, polymer-based systems^6^. Biopolymers with their accessibility, cost fairness, low toxic nature, biodegradability and biocompatibility are well-known precursors to form an eco-friendly adsorption system^28^. Natural polymers tend to make three-dimensional (3D) networks via cross-linking agents. Various polypeptides^29^, and polysaccharides, including cellulose^30^, β-cyclodextrin^31^, agar^32^, arabic gum^33^, etc. have been used in hydrogel nanocomposites with eye-catching properties lying on their structural flexibility, facile functionalization and modifications for the desired purposes^34^. Apart from the 3D network of the cross-linked natural polymer-based hydrogels, which can enhance the pollutant’s entrapment, the functional groups on the hydrogel have direct interactions with contaminants to improve the adsorption efficiency^35–38^. In connection with this, pectin is a biopolymer with high biocompatibility and biodegradability utilized in different facets, including drug delivery, tissue engineering, and adsorption^39,40^. Due to the nontoxic nature of the pectin biopolymer, it is also employed as edible fibers. These pectin-containing fibers have the role of a barrier against gas and oil molecules or as active material’s carrier, like dyes, odors, and antioxidants^41^. Moreover, the poor mechanical strength and low adsorption efficiency and low processability of the individual polysaccharide-based hydrogels could be ameliorated by various substances, including inorganic nanomaterials, mesoporous siliceous, and carbonaceous substances^14^.

As preventing adsorbent loss during its isolation from the reaction mixture matters, preparing magnetic nanocomposites or applying natural substances with innate magnetic feature has been noticed^42^. In this regard, magnetic nanoparticles (MNPs) integration in the nanocomposites barricade the nanocomposite loss during adsorption–desorption cycles and improves the scale-up efficiency in water refinement and adsorption of contaminants^43,44^. The incorporation of clay-based materials, i.e., bentonite in the adsorbent system has lighten the way of adsorption capacity improvement whether in single-phase or as a composites’ component^45^. Bentonite renders enhanced surface reactivity, ion-exchange features, flexibility, nontoxicity, biocompatibility, and high surface area leading to an improved adsorption capacity^46^. Lately, many studies displayed the impressive effect of bentonite layers incorporation between natural polymer-based hydrogels and investigated their impact on the structural physicochemical characteristics in adsorption process^47,48^. For example, bentonite layers intercalation with polyaniline polymeric chains led to developing a product with increased surface area, high conduction, mechanical stability, and elevated adsorption capacity^49,50^. Furthermore, several reports have indicated the bentonite’s beneficial physicochemical features induced to the nanoadsorbent structure by integrating bentonite and many metal oxides or metal-based structures^51,52^.

**Figure 1:**
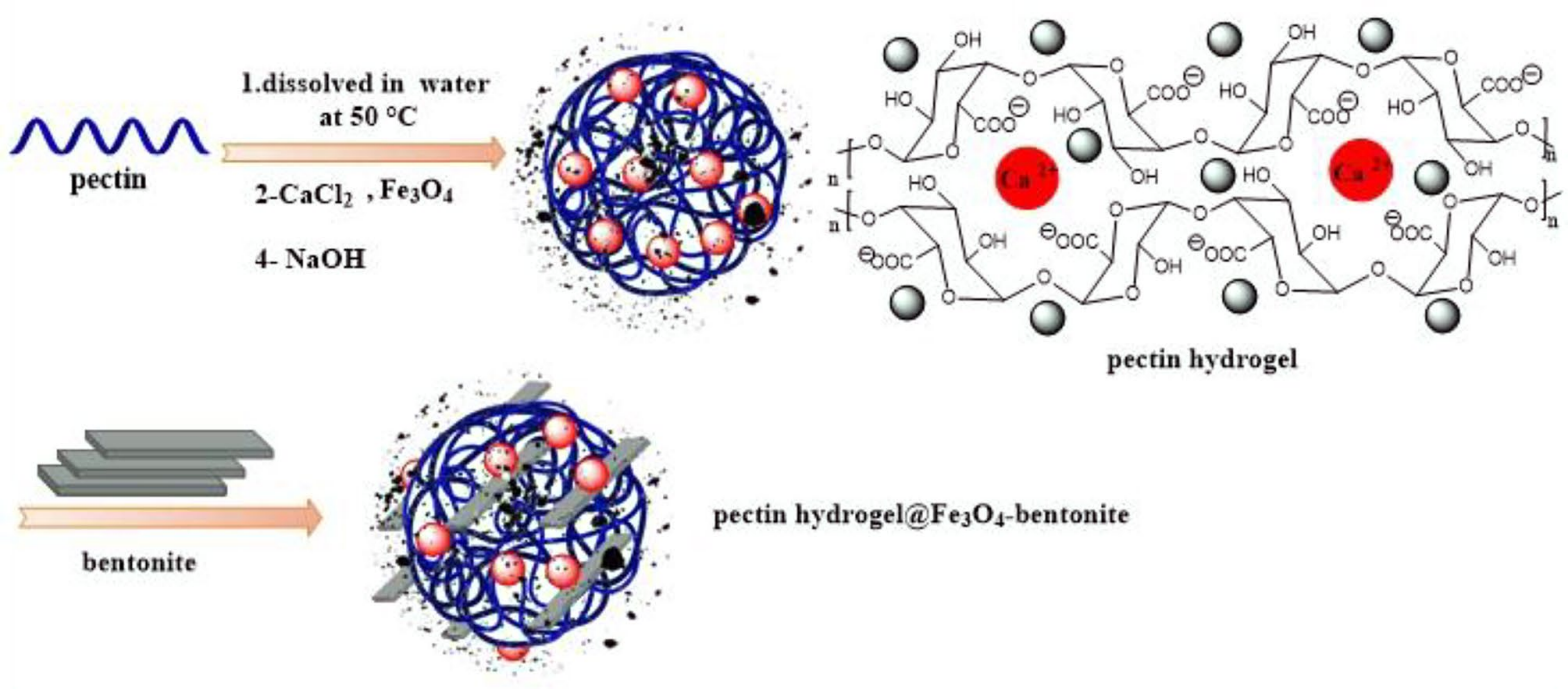
The scheme of pectin hydrogel@Fe_3_O_4_-bentonite magnetic nanocomposite preparation pathway.

Herein, a novel nanoadsorbent was prepared based on natural cross-linked pectin hydrogel with calcium chloride (CaCl_2_) cross-linking agent via an ionic cross-linking process. Then, it was magnetized with the as-prepared nanoscale Fe_3_O_4_ MNPs with a spherical morphology to be conveniently separated from the reaction mixture. Afterward, bentonite clay was introduced to the adsorbent system to elevate the surface area and porosity. The privileges of prepared pectin hydrogel@Fe_3_O_4_-bentonite nanoadsorbent are its economic production procedure from natural and inexpensive precursors, the high surface area of 68.904 m^2^.g^−1^ as a result of ion cross-linking of pectin hydrogel and the addition of bentonite to the structure with 100.011 m^2^.g^−1^ surface area, abundant reactive sites, facile magnetic recovery, as well as its reusability in three adsorption–desorption runs with no remarkable decrease in the yield of the adsorption reaction. This nanoadsorbent obeys the Freundlich isotherm and the pseudo-second-order model for the adsorption process and kinetics of the description, respectively. Also, by the acquired results from the adsorption process, the prepared nanoadsorbet exhibited a high adsorption capacity of 833.333 mg/g and 909.091 mg/g for CPF and CV adsorption from an aqueous media in an optimum condition, respectively. Based on the experimental data, the pectin hydrogel@Fe_3_O_4_-bentonite nanoadsorbent rendered a desirable performance in organophosphorus pesticide (CPF) and organic cationic dye (CV) adsorption. The current study exhibits a novel, affordable, eco-friendly, promising adsorbent with a low-cost inorganic cross-linker, advanced adsorption capacity, and high surface area for effective dye removal and dephosphorization from water. The preparation steps are depicted in Fig. 1.

## Experimental section

### Material

High-purity materials, including Pectine (Sigma-Aldrich Cas.no, (9000–69-5)), Bentonite (Sigma-Aldrich Cas.no, (1302–78-9)), Ferric chloride hexahydrate (FeCl_3_·6H_2_O) (ACS reagent, 97%), Ferrous chloride tetrahydrate (FeCl_2_·4H_2_O) (Reagent Plus^®^, 98%, Sigma-Aldrich), Ethanol (96%, Sigma-Aldrich), Sulfuric acid (ACS reagent, 95.0–98.0%, Sigma-Aldrich), Diethyl ether (ACS reagent, anhydrous, ≥ 99.0%, contains BHT as an inhibitor, Sigma-Aldrich), Ammonia (puriss., anhydrous, ≥ 99.9%, Sigma-Aldrich), Sodium hydroxide (reagent grade, ≥ 98%, pellets (anhydrous), Sigma-Aldrich), Calcium chloride (CaCl_2_)( anhydrous, powder, 99.99% trace metals basis).

### Instruments

The chemical solvents and reagents applied in this work, viz., FeCl_3_.6H_2_O, FeCl_2_.4H_2_O, distilled water, ammonia solution (25%), acetone, pectin, calcium chloride, methanol, sodium hydroxide, bentonite, and ethanol were provided by Sigma-Aldrich Company, USA. Based on the preparation process of the pectin hydrogel@Fe_3_O_4_-bentonite nanoadsorbent, some analytical and spectroscopic experiments have been taken to authenticate the structural features in every preparation stage as follows. Fourier-transform infrared (FT-IR) spectra by Shimadzu FTIR-8400S model, Japan, apparatus using KBr pellet. The nanoadsorbent’s elemental detection was executed by energy-dispersive x-ray analysis (EDX) (VEGA-TESCAN-XMU model, Czech Republic). The X-ray diffractometer (DRON-8, Saint-Petersburg, Russia) was utilized to record the prepared samples’ X-ray diffraction (XRD) pattern. The morphology of the samples was perused by the Hitachi S-5200 apparatus, Japan, and ZEISS SIGMA, Germany, for the field emission scanning electron microscope (FESEM) investigation. The study of the sample’s magnetic behavior was accomplished by VSM analysis (Meghnatis, daneshpajooh Kashan, Iran). Also, the BAHR-STA 504 apparatus, Germany, was employed to investigate the change in the weight of the samples with temperature enhancement by thermogravimetric analysis (TGA) under an argon atmosphere. The samples’ specific surface area, pore width, and pore volume were determined by the Brunauer–Emmett–Teller (BET, Micrometics ASAP2020, USA). The samples’ surface charge was determined by zeta potential (Bruker, D8 advance, USA). Moreover, the analyses were executed to specify the properties and considerable adsorption performance of the nanoadsorbent.

### Preparation of the magnetic mesoporous nanoadsorbent

#### Preparation of the Fe_3_O_4_ magnetic nanoparticles

Co-deposition approach was applied to prepare Fe_3_O_4_ MNPs. Initially, 2.35 g of FeCl_3_.6H_2_O and 0.86 g FeCl_2_.4H_2_O, along with 40.0 mL of distilled water, were magnetically stirred under nitrogen gas flow at 80 °C for 30 min. Then, 15.0 mL of ammonia was added to the suspension to form Fe_3_O_4_ MNPs. Ultimately, the black precipitate was magnetically collected and rinsed with distilled water several times; then, it was dried at 70–80 °C for 12 h.

#### Preparation of the pectin hydrogel

Pectin (0.75 g) was dissolved in 75.0 mL of distilled water at 50 °C for 30 min. Then, 1.5 g of CaCl_2_ with 30.0 mL of distilled water was injected dropwise and stirred for 40 min. Afterward, 30.0 mL ethanol was added. 0.12 g of sodium hydroxide with 15.0 mL of distilled water was poured dropwise and mixed for 30 min, and the pectin hydrogel was formed. It was taken into the ice bath to fix its gelly nature. After forming and hardening the gel, the reaction solution was washed with 50.0 mL of ethanol and 50.0 mL of distilled water.

#### Preparation of the magnetic pectin hydrogel@Fe_3_O_4_

First, 0.25 g of pectin hydrogel was dissolved in 50.0 mL of distilled water for 30 min at 50 °C. Afterward, 0.15 g of CaCl_2_ was slowly poured into the reaction mixture and stirred for 30 min at room temperature. 0.35 g of the as-prepared Fe_3_O_4_ MNPs were added into 20.0 mL of distilled water and dispersed via sonication for 40 min^53^. Next, it was added gently to the reaction solution in 2–3 steps and stirred for 30 min. Eventually, 10.0 mL of methanol, 0.04 g of sodium hydroxide, and 5.0 mL of distilled water were added to the mixture and stirred for 1 h. After performing the mentioned steps, the reaction solution was washed many times with distilled water and ethanol, and a magnetic hydrogel was obtained. The obtained magnetic hydrogel was freeze-dried for 24 h. The pectin hydrogel@Fe_3_O_4_ magnetic hydrogel was prepared by an ionic cross-linking procedure. The utilization of Ca^2+^ divalent cation as a cross-linking agent in this preparation helped to create a 3D structure. The carboxylic acid functional groups on the pectin polymeric chain changed into COO^-^ in an alkaline peripheral caused by NaOH. Also, the hydroxyl groups became O^-^. These anions have electrostatic interactions with Ca^2+^. Finally, the precipitate was formed by applying ethanol and diluted NaOH solution.

#### Preparation of the magnetic pectin hydrogel@Fe_3_O_4_-bentonite nanoadsorbent

For the preparation of pectin hydrogel@Fe_3_O_4_-bentonite magnetic nanoadsorbent, initially, 1.0 g bentonite was added in 100.0 mL of distilled water and remained for 2 h at an ambient temperature. Then, pectin hydrogel@Fe_3_O_4_ (0.25 g) was dispersed in 25.0 mL of distilled water for 20 min without temperature via ultrasonication. Bentonite suspension was added to the pectin hydrogel@Fe_3_O_4_ solution and dispersed ultrasonically for another 20 min. In the last step, the obtained solution was stirred at 25 °C for 16 h. Finally, the obtained particles were magnetically separated, rinsed with distilled water and ethanol, and dried in a vacuum oven for 24 h. In this preparation stage, bentonite attaches to the surface of pectin hydrogel@Fe_3_O_4_ magnetic nanocomposite from the silicon head, and the pectin hydrogel@Fe_3_O_4_-bentonite magnetic nanoadsorbent was formed. Ultimately, ethanol and diluted NaOH solution were utilized to form the precipitate, as shown in Fig. 1.

### Batch adsorption experiments

Experimental investigations were performed to peruse pectin hydrogel@Fe_3_O_4_-bentonite magnetic nanocomposite capability for CPF and CV adsorption from aqueous media.

The variable parameters were perused to calculate the adsorption capacity, like pH, adsorbent amount, contact time, and initial concentration of CPF and CV. To investigate the optimum adsorption conditions, various parameters were examined. For pH adjustment in the range of 4 to 9, 0.1 M of HCl and 0.1 M of NaOH were utilized, and 5–25 mg of the pectin hydrogel@Fe_3_O_4_-bentonite nanoadsorbent was applied in the 5–25 min contact time, and the initial concentrations of the CPF and CV were 50–400 ppm. Besides, the adsorption isotherms were perused by comparing the experimental results and Freundlich, Langmuir, Temkin, and Dubinin-Radushkevich (D-R) models. Furthermore, the adsorption kinetics were investigated via pseudo-first-order, pseudo-second-order, and Elovich adsorption kinetic models. The concentrations of CPF and CV were determined by a UV–Vis spectrophotometer. In addition, the CPF and CV’s adsorption capacity by the pectin hydrogel@Fe_3_O_4_-bentonite nanoadsorbent was measured via Eq. (1) and Eq. (2), respectively^6^.

1 $$\mathrm{R }(\%) = \frac{({C}_{0}-{C}_{i})}{{C}_{0}}\times 100$$

2 $$\mathrm{Qe} = \frac{({C}_{0}-{C}_{i})\times V}{m}$$

where C_i_ (mg/L) is the initial concentration and C_e_ (mg/L) is an equilibrium concentration of CPF and CV in an aqueous solution. V (L) stands for the solution volume. And m (g) represents the pectin hydrogel@Fe_3_O_4_-bentonite nanoadsorbent’s weight.

### Regeneration and retrievability

The retrievability assessment was executed to give a view of the regeneration potential of the pectin hydrogel@Fe_3_O_4_-bentonite nanoadsorbent and the water treatment procedure expenses. In this study, three consecutive reuse cycles in optimum conditions were carried out to observe the pectin hydrogel@Fe_3_O_4_-bentonite nanoadsorbent’s regeneration after CPF and CV adsorption. The desorption experiment of CPF was executed as follows. After CPF adsorption by pectin hydrogel@Fe_3_O_4_-bentonite nanoadsorbent in optimum conditions, i.e., 10.0 mL of solution volume, 0.005 g of adsorbent dosage, 20 min contact time, solution pH of 7, 300.0 mg/L initial concentration at an ambient temperature, the nanoadsorbent was poured into 10.0 mL ethanol and stirred at 25 °C for 3 h. Moreover, for the CV desorption experiment, after CV adsorption by pectin hydrogel@Fe_3_O_4_-bentonite nanoadsorbent in optimum conditions, viz., 10.0 mL of solution volume, 0.005 g of adsorbent dosage, 15 min contact time, solution pH of 9, 300.0 mg/L initial concentration at an ambient temperature, the nanoadsorbent was added into a HCl solution (0.1 M) and stirred at 25 °C. After desorption, the nanoadsorbent was magnetically isolated from the mixture. The released CPF and CV amount were then investigated through a UV–Vis spectrophotometer. The desorption percentage (D%) was calculated using Eq. (3).

3 $$\mathrm{D} (\%) =\frac{A}{B}\times 100$$

where A (mg) belongs to the desorbed contaminant quantity in the elution medium, and B (mg) stands for the adsorbed contaminant quantity by pectin hydrogel@Fe_3_O_4_-bentonite nanoadsorbent.

## Results and discussion

Regarding the preparation steps of the magnetic nanoadsorbent, such as Fe_3_O_4_ MNPs preparation, pectin hydrogel formation, *ex-situ* magnetization of pectin hydrogel by adding the as-prepared Fe_3_O_4_ MNPs (pectin hydrogel@Fe_3_O_4_), and bentonite incorporation into the composite (pectin hydrogel@Fe_3_O_4_-bentonite), different characterizations were employed to investigate the prepared nanoadsorbent’s features as follows.

### Preparation of the magnetic pectin hydrogel@Fe_3_O_4_-bentonite nanoadsorbent

Pectin hydrogel@Fe_3_O_4_-bentonite magnetic nanocomposite was prepared and applied as an efficient nanoadsorbent for CPF and CV removal from aqueous media. As demonstrated in Fig. 1, preparing this nanoadsorbent is comprised of Fe_3_O_4_ MNPs preparation via a co-sedimentation approach, pectin hydrogel preparation via employing the Ca^2+^ divalent cation as a cross-linker, and ultimately, bentonite as a clay material was added to the composite to enhance the surface area. As stated, due to their cross-linked 3D structure, hydrogels render high porosity leading to higher CPF and CV adsorption via the physical capturing of contaminants into the pores. On the other hand, diverse functional groups on the hydrogel, i.e., hydroxyl and carboxyl groups, result in hydrogen bonding and electrostatic interactions due to the CPF and pectin heteroatom structures.

However, the main interaction between the pectin hydrogel@Fe_3_O_4_-bentonite nanoadsorbet with negative surface charge (based on the data reported in Table 2) and CV cationic dye is electrostatic interactions. Therefore, the magnetic pectin hydrogel@Fe_3_O_4_-bentonite nanoadsorbet with heteroatom structures and clay substances led to an improved organic dye and organophosphorus pesticide adsorption capacity.

### Characterization of the prepared magnetic pectin hydrogel@Fe_3_O_4_-bentonite nanoadsorbent

#### Fourier transform infrared spectroscopy

**Figure 2:**
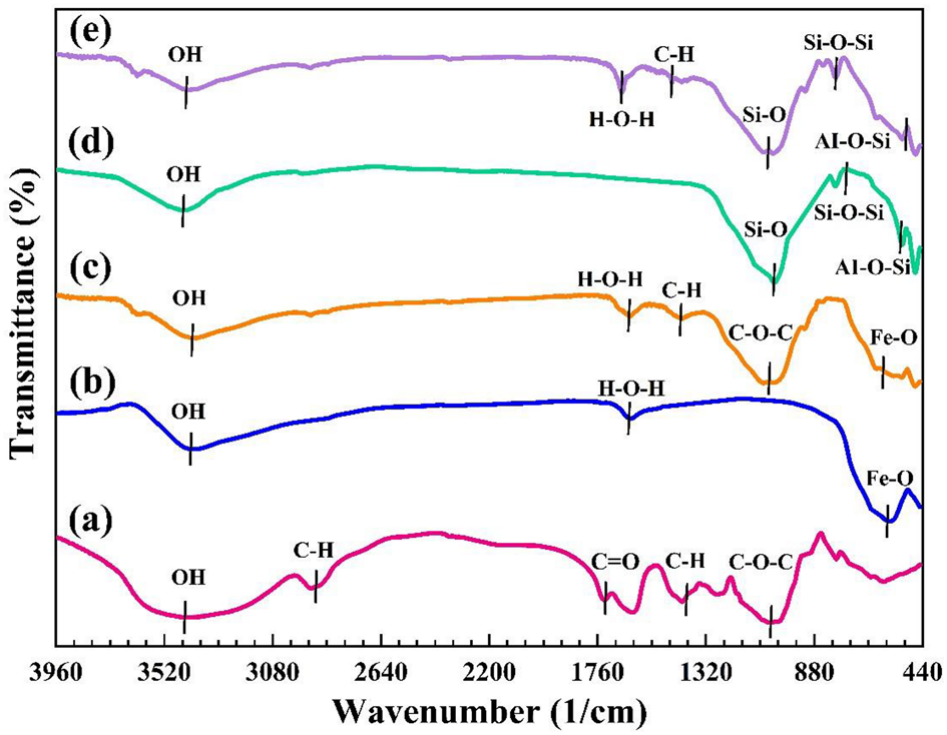
The FT-IR spectra of (**a**) Pectin hydrogel, (**b**) Fe_3_O_4_ MNPs, (**c**) Pectin hydrogel@Fe_3_O_4_, (**d**) bentonite (**e**) Pectin hydrogel@Fe_3_O_4_-bentonite.

Fourier transform infrared spectroscopy (FTIR) is one of the most suitable and conventional techniques for the qualitative identification of the structural functional groups of species and for determining the structure of different molecules, especially organic species. Figure 2 shows the FTIR spectra of pectin hydrogel, Fe_3_O_4_ MNPs, pectin hydrogel@Fe_3_O_4_, bentonite, and pectin hydrogel@Fe_3_O_4_-bentonite, respectively. As depicted in the FTIR spectrum of the pectin hydrogel (Fig. 2a), the formation of the pectin hydrogel is confirmed by the presence of five distinctive absorption peaks. The broad absorption peak that arose at 3425 cm^−1^ is correlated with the –OH groups’ stretching vibration in hydrogen bonding^54^. Two sharp peaks at 2917 cm^-1^ and 1425 cm^-1^ are due to the C–H stretching and bending vibrations, respectively. Furthermore, the stretching vibrations of –C–O–C in the glycosidic bond and carbonyl group in –COOCH_3_ emerge at 1049 cm^-1^ and 1736 cm^-1^, respectively^42,55^. As observed in Fig. 2b, the spectrum of Fe_3_O_4_ has three characteristic absorption peaks. The vibration peak at 570 cm^−1^ is related to the bonds between iron and oxygen (Fe–O)^6,56^. The H–O–H vibration has an absorption peak in the region of 1624 cm^−1^^57^. Besides, The band at 3403 cm^−1^ corresponds to the hydroxyl groups’ stretching vibration providing a hydrophilic component for Fe_3_O_4_ MNPs and increasing their dispersity^58^. In the pectin hydrogel@Fe_3_O_4_ spectrum (Fig. 2c), all peaks are associated with the pure Fe_3_O_4_ NPs and pectin hydrogel. As debated above, the broad absorption peak at 3410 cm^−1^ corroborates the stretching vibration of the –OH hydrogen bond observed in both pure substances. The peaks at 2920 cm^-1^ and 1425 cm^−1^ confirm the pectin hydrogel’s C–H stretching and bending vibrations, respectively. The peak at 1626 cm^−1^ is attributed to the H–O–H bond of Fe_3_O_4_ MNPs. The pectin hydrogel’s stretching vibrations of the –C–O–C group in the glycosidic bond and C = O in –COOCH_3_ arose at 1049 cm^−1^ and 1736 cm^−1^, respectively. Also, a distinguished peak at 560 cm^−1^ is allocated to the vibrations of the Fe–O bond in Fe_3_O_4_ MNPs^31,59,60^. The presence of all peaks indicates the formation of pectin hydrogel@Fe_3_O_4_ nanocomposite.

Figure 2d represents the bentonite spectrum which resembles amorphous silica, with absorption peaks at 1040 cm^−1^, 794 cm^−1^, 524 cm^−1^, and 473 cm^−1^, caused by stretching and bending vibrations of SiO_4_^2^. The Si–O bonds are evident in the silicate structure and are easily identified in the FTIR spectrum by strong absorption peaks in the 1000–1100 cm^−1^ region. In contrast, 473 cm^−1^ and 524 cm^−1^ peaks are caused by Si–O-Al and Si–O-Si bending vibrations, respectively. In addition, the broad absorption peak at 3440 cm^−1^ is due to the stretching of the –OH hydrogen bond, which corresponds to the frequencies of –OH (silanol group (Si–O–H))^61,62^.

Figure 2e demonstrates the FTIR spectrum of the prepared pectin hydrogel@Fe_3_O_4_-bentonite nanoadsorbent. When the pectin hydrogel@Fe_3_O_4_ was functionalized with bentonite binder, the pectin hydrogel@Fe_3_O_4_ became more reactive, some changes were caused in the peaks of pectin hydrogel@Fe_3_O_4,_ and new absorption peaks appeared. The peak that arose at 3420 cm^−1^ is assigned to the stretching vibration of the –OH, which was observed in all three pure substances. The adsorption peak at 1662 cm^−1^ is allocated to the vibrations of H–O–H in the Fe_3_O_4_ MNPs. The bands observed at 2922 cm^−1^ and 1420 cm^−1^ are due to C–H stretching and bending vibrations^63^. The peak at 1043 cm^−1^ corresponds to the stretching vibrations of –C–O–C (glycosidic bond) of the –COOCH_3_ group; all these peaks are related to pectin hydrogel. As mentioned above, the absorption bonds at 1038 cm^−1^, 794 cm^−1^, and 470 cm^−1^ are due to stretching and bending vibrations (Si–O-Si), belonging to four-dimensional SiO_4_^2^, which is very evident in bentonite structure. Furthermore, the 524 cm^−1^ peak is caused by Al–O–Si bending vibrations, which are all specific to bentonite material. Ultimately, by combining all the distinctive absorption peaks and the bonding of materials with unique functional groups with a new chemical structure, the prepared magnetic nanoadsorbent was formed.

#### Energy dispersive X-ray spectroscopy

**Figure 3:**
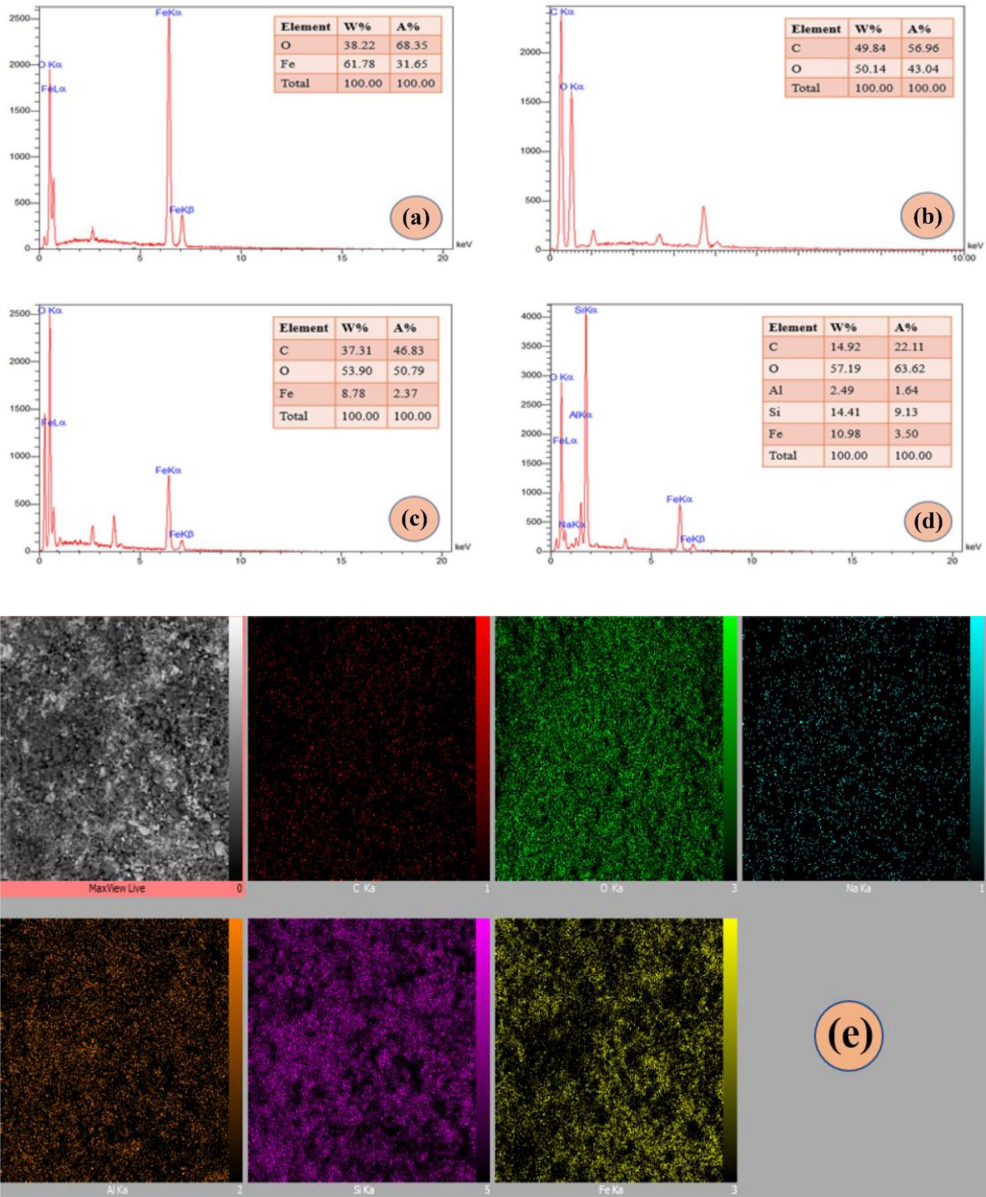
EDX analysis of (**a**) Fe_3_O_4_ MNPs, (**b**) Pectin hydrogel, (**c**) Pectin hydrogel@Fe_3_O_4_, (**d**) Pectin hydrogel@Fe_3_O_4_-bentonite nanoadsorbent, and (**e**) EDX mapping of Pectin hydrogel@Fe_3_O_4_-bentonite nanoadsorbent.

For the investigation of the sample’s elements and elemental distribution in the sample, the EDX analysis was executed (Fig. 3). The O and Fe elements are assigned to the Fe_3_O_4_ superparamagnetic MNPs with 38.22 W% and 61.78 W%, respectively (Fig. 3a). Based on the EDX spectrum in Fig. 3b, two distinctive C and O elements represent pectin hydrogel, showing 49.84 W% and 50.14 W% due to the quantitative results. The integration of Fe_3_O_4_ MNPs and pectin hydrogel was successfully confirmed via the presence of the Fe element (8.78 W%) in the pectin hydrogel spectrum (Fig. 3c). According to adding Al (2.49 W%), Si (14.41 W%), and O (57.19 W%), indicating the bentonite structural elements, the formation of the final pectin hydrogel@Fe_3_O_4_-bentonite is authenticated (Fig. 3d). Furthermore, Fig. 3e depicts the well-distribution of the elements in the magnetic pectin hydrogel@Fe_3_O_4_-bentonite nanoadsorbent.

#### Field emission scanning electron microscopy

**Figure 4:**
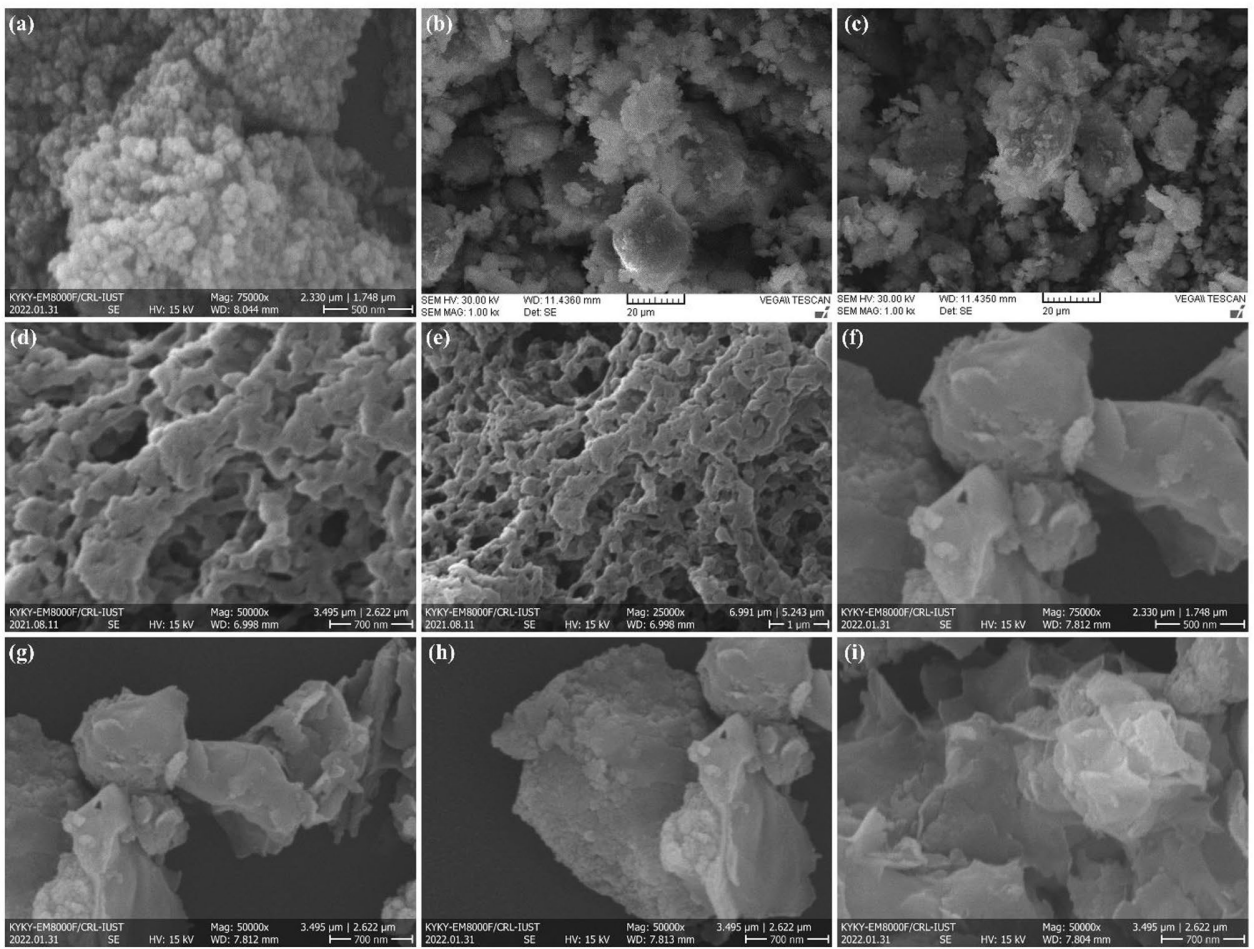
FESEM images of (**a**) Fe_3_O_4_ magnetic nanoparticles, (**b,c**) Bentonite, (**d,e**) Pectin hydrogel@Fe_3_O_4_, (**f–i**) Pectin hydrogel@Fe_3_O_4_-bentonite nanoadsorbent.

The FESEM images were provided to clarify and investigate the samples’ morphology, size, shape uniformity, distribution of particle size, and possible agglomerations. Figure 4a displays the FESEM image of the prepared Fe_3_O_4_ MNPs. Although the nanoparticles have a generally uniform size and spherical morphology, they are aggregated in some areas. Besides, the prepared nanoparticles rendered a narrow size distribution of ca. 40–70 nm, participating in the final adsorbent to form the Pectin hydrogel@Fe_3_O_4_-bentonite nanoadsorbent. Based on the FESEM images in Fig. 4b,c, the bentonite components demonstrated bullet-like morphology and porous structure. The size and molecular mass of bentonite’s structural phases were not alike; the material’s size was different. The FESEM images of the Pectin hydrogel@Fe_3_O_4_ have been taken in 700 nm and 1 μm magnifications, as shown in Fig. 4d,e, respectively. The spherical Fe_3_O_4_ MNPs were well placed on the pectin hydrogel substrate, functionalized, and a magnetic hydrogel was formed. However, since the ion cross-linking of the pectin polymer strings was performed in the presence of the as-prepared Fe_3_O_4_ MNPs, these MNPs are not simply visible in the SEM images of the pectin hydrogel@Fe_3_O_4_-bentonite nanoadsorbent, and the hydrogel network covers them. From the comparison of the FESEM images of the pectin hydrogel@Fe_3_O_4_-bentonite nanoadsorbent and pectin hydrogel@Fe_3_O_4_ nanocomposite, the modifications made on the surface in order to apply the nanocomposite with better efficiency in water contaminant and chlorpyrifos poison absorption. In Fig. 4f–i, adding bentonite exhibited significant alterations on the surface of the magnetic hydrogel, including smoothing the pores’ surface and forming a layered structure.

#### Vibrating-sample magnetometer (VSM) study

**Figure 5:**
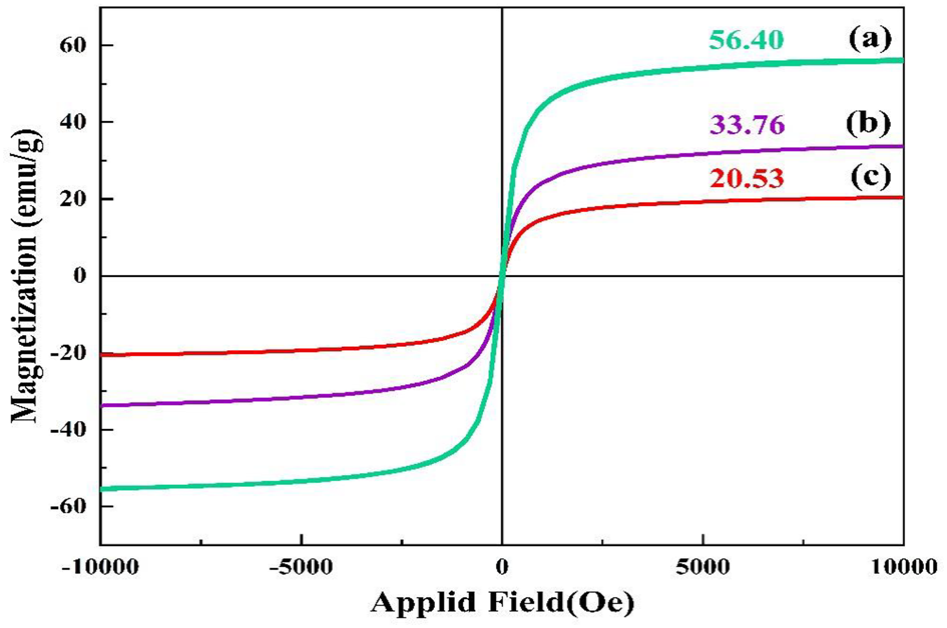
VSM curves of (**a**) Fe_3_O_4_ MNPs, (**b**) Pectin hydrogel@Fe_3_O_4_, (**c**) Pectin hydrogel@Fe_3_O_4_-bentonite nanoadsorbent.

The magnetic characteristics of the samples, i.e., Fe_3_O_4_ MNPs, pectin hydrogel@Fe_3_O_4_, pectin hydrogel@Fe_3_O_4_-bentonite nanoadsorbent were conducted via the VSM analysis in an applied magnetic field from − 10,000 to + 10,000 Oe at 25 °C, as displayed in Fig. 5a–c. The magnetic coercivity and magnetic retentivity properties of the Fe_3_O_4_ MNPs, pectin hydrogel@Fe_3_O_4_, and pectin hydrogel@Fe_3_O_4_-bentonite nanoadsorbent are zero. Hence, the prepared samples have superparamagnetic behavior. According to the VSM curve shown in Fig. 5, the magnetic saturation of the bare Fe_3_O_4_ MNPs is 56.40 emu/g. Then, the magnetic saturation reduced from 33.76 emu/g for pectin hydrogel@Fe_3_O_4_ to 20.53 emu/g for pectin hydrogel@Fe_3_O_4_-bentonite nanoadsorbent. The observed decrease in magnetic saturation is related to adding pectin as a natural polysaccharide and bentonite to increase the surface’s active sites. Besides, these materials are non-magnetic, and reducing the magnetic saturation due to the chemical modifications and incorporation of the organic substances on the nanoadsorbent’s surface seems logical. Yet, the magnetic saturation of both pectin hydrogel@Fe_3_O_4_ and pectin hydrogel@Fe_3_O_4_-bentonite nanoadsorbents is sufficient to be isolated from the mixture by a magnet.

#### Thermogravimetric analysis

**Figure 6:**
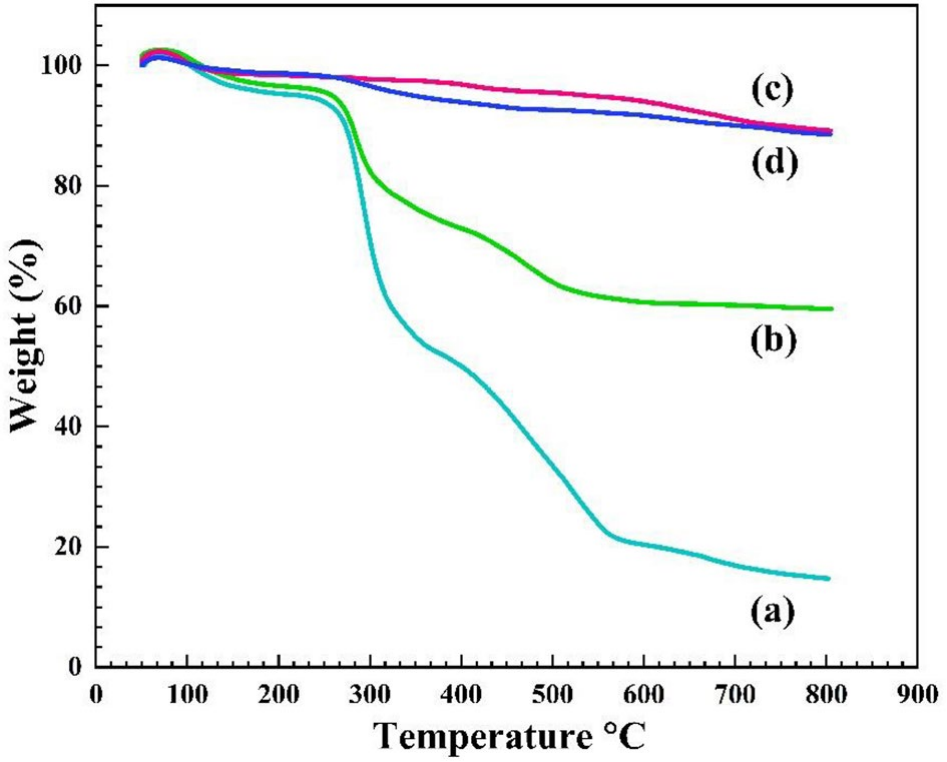
TGA curve of (**a**) pectin hydrogel, (**b**) Pectin hydrogel@Fe_3_O_4_, (**c**) bentonite, (**d**) Pectin hydrogel@Fe_3_O_4_-bentonite nanoadsorbent.

Figure 6 demonstrates the thermogravimetric behavior of the pectin hydrogel, pectin hydrogel@Fe_3_O_4_, bentonite, and pectin hydrogel@Fe_3_O_4_-bentonite nanoadsorbent, and their stability over temperature rise to 800 °C with 10 °C/min stable heating rate under an air atmosphere by the thermogravimetric analysis (TGA). The thermogravimetric behavior of the pectin hydrogel (Fig. 6a) shows a 2% weight loss at 50–223 °C, indicating the evaporation and removal of the entrapped and absorbed water molecules in the pectin hydrogel’s pores and surface. Further, increasing the temperature to 350 °C shows a 25% weight loss due to organic moieties’ separation and thermal dissociation. The thermal behavior of the pectin hydrogel shows that the degradation of pectin hydrogel starts at ca. 250 °C and continues to 350 °C^64,65^. Approximately 70% of pectin hydrogel weight decreases between 200 and 550 °C. The residual weight of pectin hydrogel at 800 °C is reported to be about 20%. As can be observed in the pectin hydrogel@Fe_3_O_4_ thermogram curve (Fig. 6b), with the temperature increase to 800 °C, ca. 67% of the weight of this sample remains, and the increase in thermal resistance compared to pectin hydrogel is only due to the formation of Fe_3_O_4_ inorganic MNPs in its substrate^66^. The observed gradual weight loss can be assigned to water evaporation from the holes in this structure as well as dehydrogenation or dehydroxylation of its surface.

According to the previously reported results, bentonite represents high thermal stability so that more than 91–95% of its weight has retained up to 700 °C^67^. Bentonite thermogram has demonstrated a continuous but partial weight loss over enhancing the temperature due to the surface’s dehydrogenation or dehydroxylation (Fig. 6c). Moreover, with a quick look at the thermogram of the pectin hydrogel@Fe_3_O_4_-bentonite nanoadsorbent in Fig. 6d, a partial weight loss (2%) in the temperature range of 150–250 °C is attributed to evaporating and removal of the absorbed water inside and on the surface of the mesoporous structure. In addition, with the increase of temperature up to 551 °C, a weight loss of about 12% occurs because of the separating and thermal decomposition of the organic moieties of the alkyl chain that covalently bonded to bentonite^68^. Also, from the weight loss difference of the three samples, ca. 21% of the pectin hydrogel@Fe_3_O_4_-bentonite nanoadsorbent’s weight consists of the organic part. As a result, it can be concluded that pectin hydrogel@Fe_3_O_4_-bentonite nanoadsorbent has high-temperature resistance.

#### X-ray diffraction analysis

**Figure 7:**
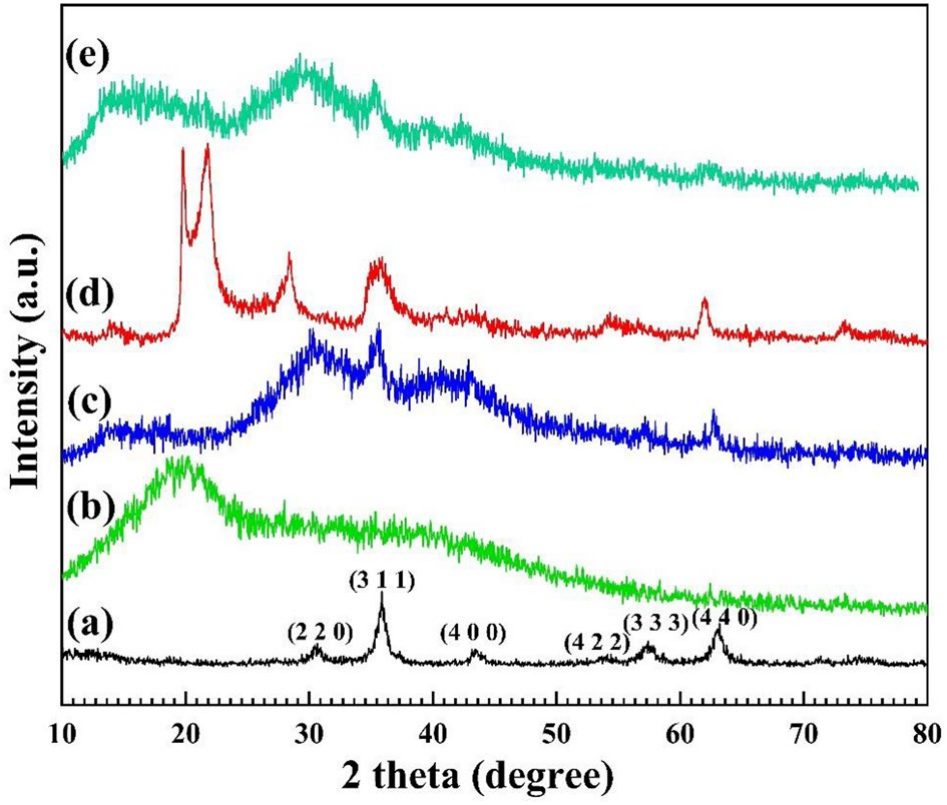
XRD pattern of (**a**) Fe_3_O_4_ MNPs, (**b**) Pectin, (**c**) Pectin hydrogel@Fe_3_O_4_, (**d**) bentonite, (**e**) Pectin hydrogel@Fe_3_O_4_-bentonite nanoadsorbent.

The crystallinity investigation of the Fe_3_O_4_ MNPs, pectin hydrogel@Fe_3_O_4_, and pectin hydrogel@Fe_3_O_4_-bentonite nanoadsorbent was carried out by X-ray diffraction (XRD) analysis from 10° to 80° (Fig. 7). The Bragg diffraction peaks originate from (1 1 1), (2 2 0), (3 1 1), (2 2 2), (4 2 2), and (5 1 1) planes in Fe_3_O_4_ MNPs, indicating cubic (fcc) structures of Fe_3_O_4_ magnetite NPs, as displayed in Fig. 7a^69^. Due to Fig. 7b, the pectin XRD pattern have an amorphous structure which is characterized by a peak emerged at 2θ = 20°^70^. According to the data reported in the articles, the XRD patterns of Fe_3_O_4_ MNPs and pectin hydrogel have strong, distinctive peaks at 2θ = 20° and 2θ = 35°, respectively^71–74^. Pectin hydrogel@Fe_3_O_4_ composite demonstrated both of these peaks at 2θ = 20° and 2θ = 35° as well, which correspond to 2θ of pectin hydrogel and Fe_3_O_4_ MNPs (Fig. 7c). A reduction in the peak intensity indicates the chemical interactions between pectin hydrogel and Fe_3_O_4_ MNPs. Moreover, based on the pattern of Fig. 7d, the crystalline peaks at 2θ = 19.76º, 21.48º, 28.52º, 35.68º, 62.16º have complied with the bentonite presence in the pectin hydrogel@Fe_3_O_4_^75^. In the XRD pattern of pectin hydrogel@Fe_3_O_4_-bentonite nanoadsorbent, a decrease in the peak intensity at 2θ = 20° and 2θ = 36° is because of the composition and related to the chemical modifications of the magnetic pectin hydrogel@Fe_3_O_4_ surface (Fig. 7e).

#### The N_2_ adsorption–desorption isotherm

**Figure 8:**
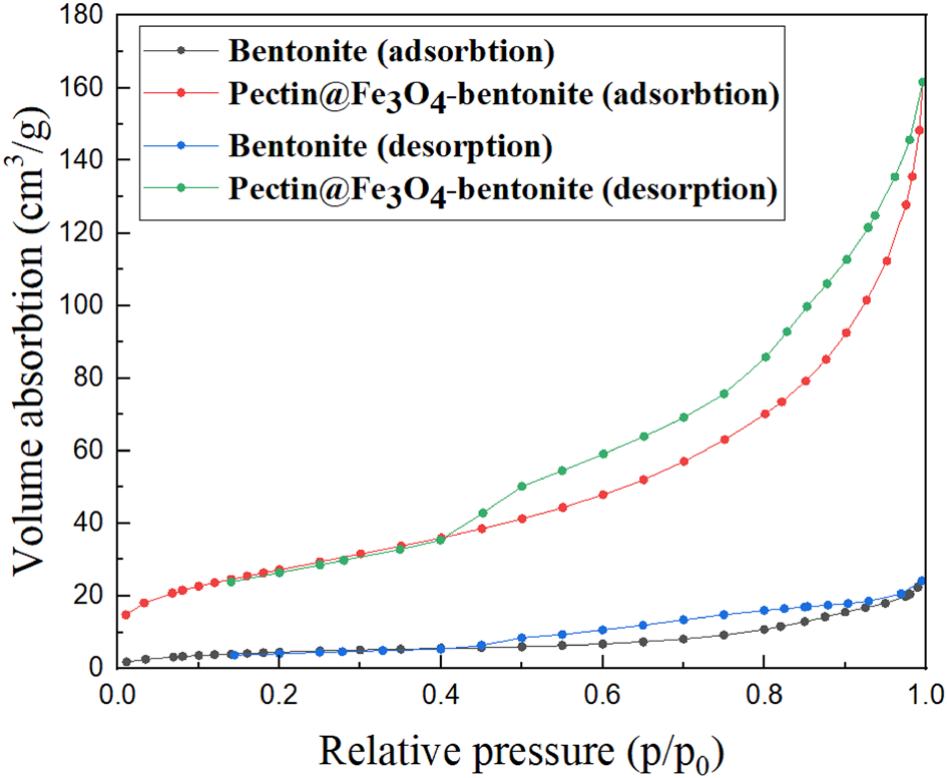
The N_2_ adsorption–desorption isotherms of bentonite and pectin hydrogel@Fe_3_O_4_-bentonite nanoadsorbent.

The BET analysis was carried out to explain the surface behavior and porosity of the bentonite and pectin hydrogel@Fe_3_O_4_-bentonite nanoadsorbent using N_2_ adsorption–desorption. As demonstrated in Fig. 8, the isotherm profiles are type-IV, representing the H4 hysteresis loop at 0.42–0.1 p/p_0_ pressure. Mesoporous materials are in the type-IV isotherm profile category based on the IUPAC categorization. According to the results, the BET surface area of the bentonite was calculated 100.011 m^2^/g, demonstrating an advanced surface area compared to the reported studies (Table 1). Furthermore, the pectin hydrogel@Fe_3_O_4_-bentonite nanoadsorbent has provided a BET surface area of 68.904 m^2^/g, which is sufficiently good. Besides, the functionalization of the pectin hydrogel, i.e., magnetization by Fe_3_O_4_ MNPs, and pectin hydrogel@Fe_3_O_4_ composition with bentonite, a reduction in the pore volume and surface area were observed. Considerably, the as-prepared pectin hydrogel@Fe_3_O_4_-bentonite nanoadsorbent demonstrating satisfactory textural features, enhanced porosity, and specific surface area could be contemplated as a potent nanoadsorbent in different water contaminant removal.

**Table 1:** Surface area, pore volume, and pore size of bentonite and pectin hydrogel@Fe_3_O_4_-bentonite nanoadsorbent.

Sample	Surface area^a^ (m^2^.g^−1^)	Pore volume^b^ (cm^3^.g^−1^)	Pore size^b^ (nm)
Bentonite	100.011	0.197	79.054
Pectin hydrogel@Fe_3_O_4_-bentonite	68.904	0.158	92.273

^a^The surface area parameter was acquired via BET analysis.

^b^The pore volume and pore size parameters were acquired via BJH analysis.

#### Zeta potential measurements

The superficial charges of the as-prepared pectin hydrogel@Fe_3_O_4_-bentonite nanoadsorbent were measured by zeta potential test. Zeta potential measurements were performed once before adsorption and once after CV dye adsorption at room temperature. Due to the reported results, at pH of 3, 5, 7, and 9, the measured zeta potential of the pectin hydrogel@Fe_3_O_4_-bentonite nanoadsorbent in deionized (DI) water were − 32.2, − 33.1, − 34.1, and − 42.8, respectively (Table 2). The increase in the absolute value of the zeta potential value is related to the pH value increase, as the structural hydroxyl and carboxylic acid groups are deprotonated. More importantly, due to the noticeable negative surface charge of the pectin hydrogel@Fe_3_O_4_-bentonite nanoadsorbent, the nanoadsorbent particles do not aggregate at various pH values. By investigating the zeta potential measurements after CV dye adsorption, it is deduced that at pH of 3, 5, 7, and 9, the pectin hydrogel@Fe_3_O_4_-bentonite nanoadsorbent’s zeta potential in the CV solution was − 32.8, − 34.3, − 35.9, and − 36.2, respectively. Since the CV dye has a cationic structure, it has robust electrostatic interactions with negatively charged pectin hydrogel@Fe_3_O_4_-bentonite nanoadsorbent.

**Table 2:** Zeta potential of the pectin hydrogel@Fe_3_O_4_-bentonite nanoadsorbent at various pH and solutions.

Sample	Solution	pH	Zeta protentional (mV)^a^
Pectin@Fe_3_O_4_-bentonite	DI water	3	− 32.2
Pectin@Fe_3_O_4_-bentonite	DI water	5	− 33.1
Pectin@Fe_3_O_4_-bentonite	DI water	7	− 34.1
Pectin@Fe_3_O_4_-bentonite	DI water	9	− 42.8
Pectin@Fe_3_O_4_-bentonite	CV	3	− 32.8
Pectin@Fe_3_O_4_-bentonite	CV	5	− 34.3
Pectin@Fe_3_O_4_-bentonite	CV	7	− 35.9
Pectin@Fe_3_O_4_-bentonite	CV	9	− 36.2

^a^The experiment was carried out at room temperature.

### Effective parameters on the adsorption of CPF and CV by magnetic pectin hydrogel@Fe_3_O_4_-bentonite nanoadsorbent

#### pH of medium

**Figure 9:**
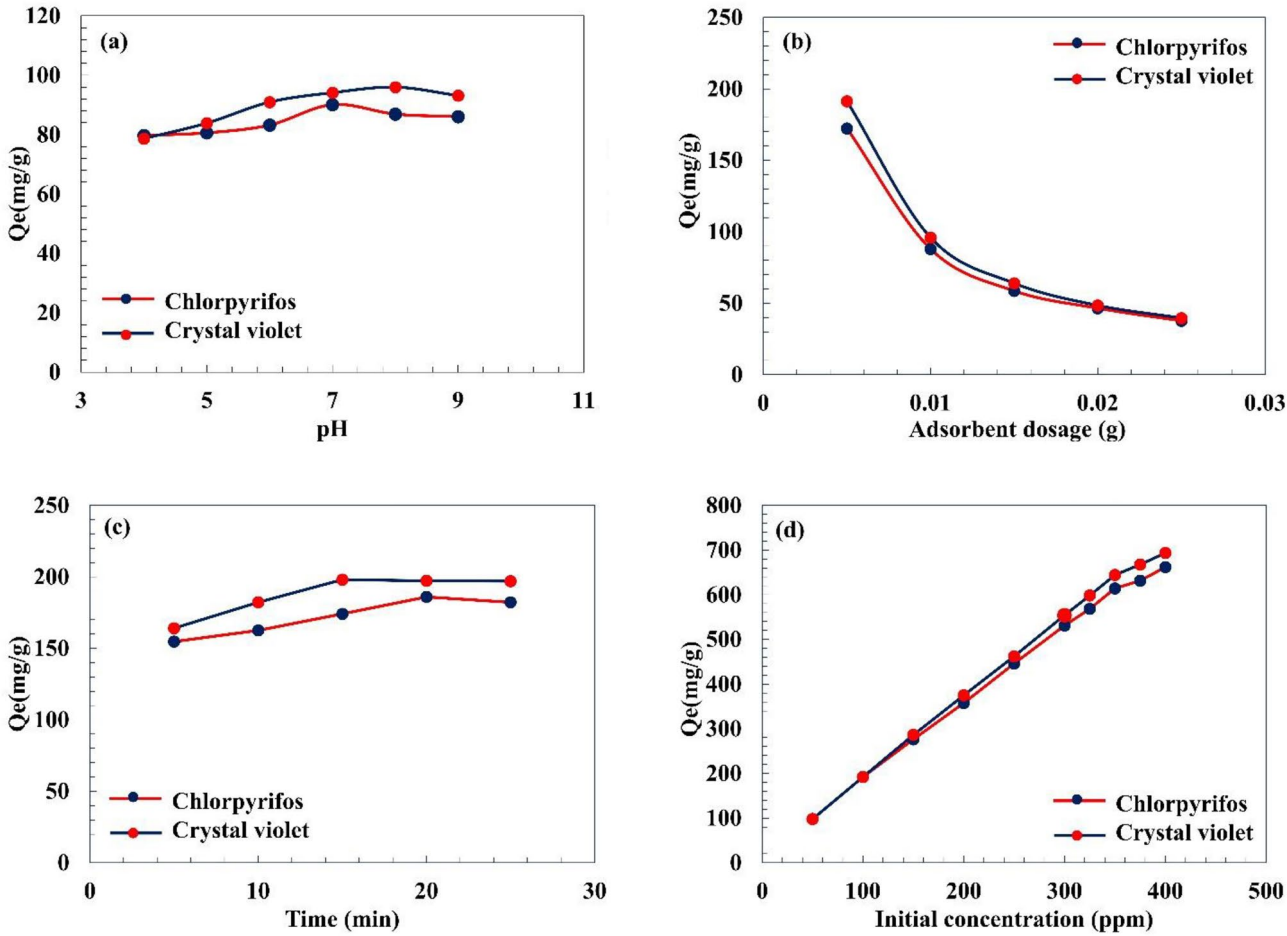
(**a**) Effect of solution pH (4–9), adsorbent dosage (0.01 g), initial concentration (100 mg/L), V (10 mL), Time (15 min), T (298 K), (**b**) adsorbent dosage (0.005–0.025 g), pH 7 and 8 for CPF and CV respectively, initial concentration (100 mg/L), V (10 mL), Time (15 min), T (298 K), (**c**) contact time (5–25 min), pH 7 and 8 for CPF and CV respectively, adsorbent dosage (0.005 g), initial concentration (100 mg/L), V (10 mL), T (298 K), (**d**) initial concentration (50–400 mg/L), pH 7 and 8 for CPF and CV respectively, adsorbent dosage (0.005 g), V (10 mL), Time (20 and 15 min for CPF and CV respectively), T (298 K).

Before optimizing various parameters, the CPF pesticide and CV dye calibration curves are provided in the supporting information file as Figs. S1 and S2, respectively. Different factors, such as the solution pH, employed adsorbent dosage, contact time, and initial concentration of the contaminant, affect the adsorption process of organic dyes and pesticides^76,77^. Further, the optimization of these effective parameters upgrades the contaminant removal. The pH effect on the CPF and CV adsorption on the pectin hydrogel@Fe_3_O_4_-bentonite nanoadsorbent is presented in Fig. 9a. Through the solution pH increase from 4 to 7, the adsorption capacity incremented from ca. 78.6 and 80.2 mg/g to 91.9 and 88.3 mg/g for CV and CPF, respectively. However, by elevating the pH to 8, the adsorption capacity of CPF was reduced to 82.6 mg/g, and the adsorption capacity of CV climbed to 96.5 mg/g. At acidic pH (i.e., pH 5), the CPF adsorption capacity declined and reached 80.6 mg/g because of the competition between the hydroxyl groups of the nanoadsorbent and excess H^+^ around the adsorbent for unoccupied adsorption sites. On the other hand, the lower CPF adsorption capacity at high pH amounts (i.e., 9) can be assigned to the electrostatic repulsion between extra OH anions and the nanoadsorbent’s OH groups^78,79^. Also, the leading bondings are the hydrogen bonding between the pectin hydrogel@Fe_3_O_4_-bentonite nanoadsorbent and CPF molecules with a non-ionic structure as an organophosphorus pesticide. Thus, the solution pH of 7 provides the minimum amount of cationic and anionic substances, diminishing their impact on the pollutant and nanoadsorbent active sites’ interactions. In this regard, for the CV with a positively-charged structure, the driving forces for its adsorption on the pectin hydrogel@Fe_3_O_4_-bentonite nanoadsorbent with negative surface charge are the electrostatic attraction, which led to an enhanced adsorption capacity at higher solution pH. This redult was consistent with Eltaweil et al. study which evinced the proper effect of the neutral and alkaline environment for adsorptive elimination of detrimental cationic dyes^80^. According to the experimental results, an ideal solution pH for CPF and CV was 7 and 8, respectively, representing the largest adsorption capacity.

#### Adsorbent dosage

The relation between the nanoadsorbents amount and the related adsorption capacity for CPF and CV was investigated using various pectin hydrogel@Fe_3_O_4_-bentonite nanoadsorbent amounts at optimum pH. Based on the results in Fig. 9b, with an increase in the adsorbent dosage from 0.005 g to 0.025 g, the adsorption capacity of the magnetic nanoadsorbent for CPF and CV decreased from ca. 170.2 mg/g and 192.3 mg/g to 37.5 mg/g and 38.1 mg/g, respectively. The availability of CPF and CV with a large amount to be adsorbed by pectin hydrogel@Fe_3_O_4_-bentonite nanoadsorbent corresponds to the enhanced adsorption capability at lower adsorbent dosages, which is associated with the increased contaminant amount available for the nanoadsorbent. Therefore, 0.005 g of pectin hydrogel@Fe_3_O_4_-bentonite nanoadsorbent was demonstrated to have the most efficient adsorbent dosage for further studies.

#### Contact time

The contact time impact on the pectin hydrogel@Fe_3_O_4_-bentonite nanoadsorbent to adsorb CPF and CV was investigated from 5 to 25 min at optimum pH and adsorbent dosage. In this regard, the CPF adsorption capacity demonstrates an increasing trend of ca. 200.0 mg/g by the progress of the time to 15 min (Fig. 9c). However, the adsorption capacity decreases slightly to 20 min and has an approximately steady trend to 25 min. The adsorption capacity of CV shows improvement to ca. 178.8 mg/g after 20 min contact time and gradually reduces to 171.6 mg/g after 25 min. Thus, an optimum contact time for CPF and CV was ascertained as 15 min and 20 min, respectively. The increasing trend at the initiation of the adsorption reaction is related to the numerous unoccupied active sites of the pectin hydrogel@Fe_3_O_4_-bentonite nanoadsorbent available for interaction with CPF organophosphorus pesticide and CV organic dye, resulting in a quick mass transfer. Efficient interactions among the nanoadsorbent’s functional groups and CPF pesticide and CV dye are progressive until it reaches the maximum equilibrium adsorption capacity after passing 15 min and 20 min of the reaction process. In contrast, no progress in adsorption capacity was observed after the optimum contact time, which is assigned to occupying the nanoadsorbent’s active sites after reaching the maximum equilibrium adsorption capacity.

#### Initial pollutant concentration

The employed contaminant concentration was the last parameter debated in this section to determine its effect on the adsorption capacity of the pectin hydrogel@Fe_3_O_4_-bentonite nanoadsorbent through regulating the CPD and CV initial concentration from 50 to 400 mg/L at optimum pH, adsorbent dosage, and contact time. Based on Fig. 9d, increasing the CPF and CV initial concentrations from 50 to 400 mg/L enhanced the adsorption capacity to 703.8 mg/g and 665.4 mg/g, respectively. Certainly, by increasing the CPF and CV initial concentration at a constant adsorbent dosage, the CPF organophosphorus pesticide and CV dye adsorbate amount to pectin hydrogel@Fe_3_O_4_-bentonite nanoadsorbent ratio was enhanced, leading to an improved adsorption capacity.

### Adsorption isotherm and adsorption kinetics studies

**Figure 10:**
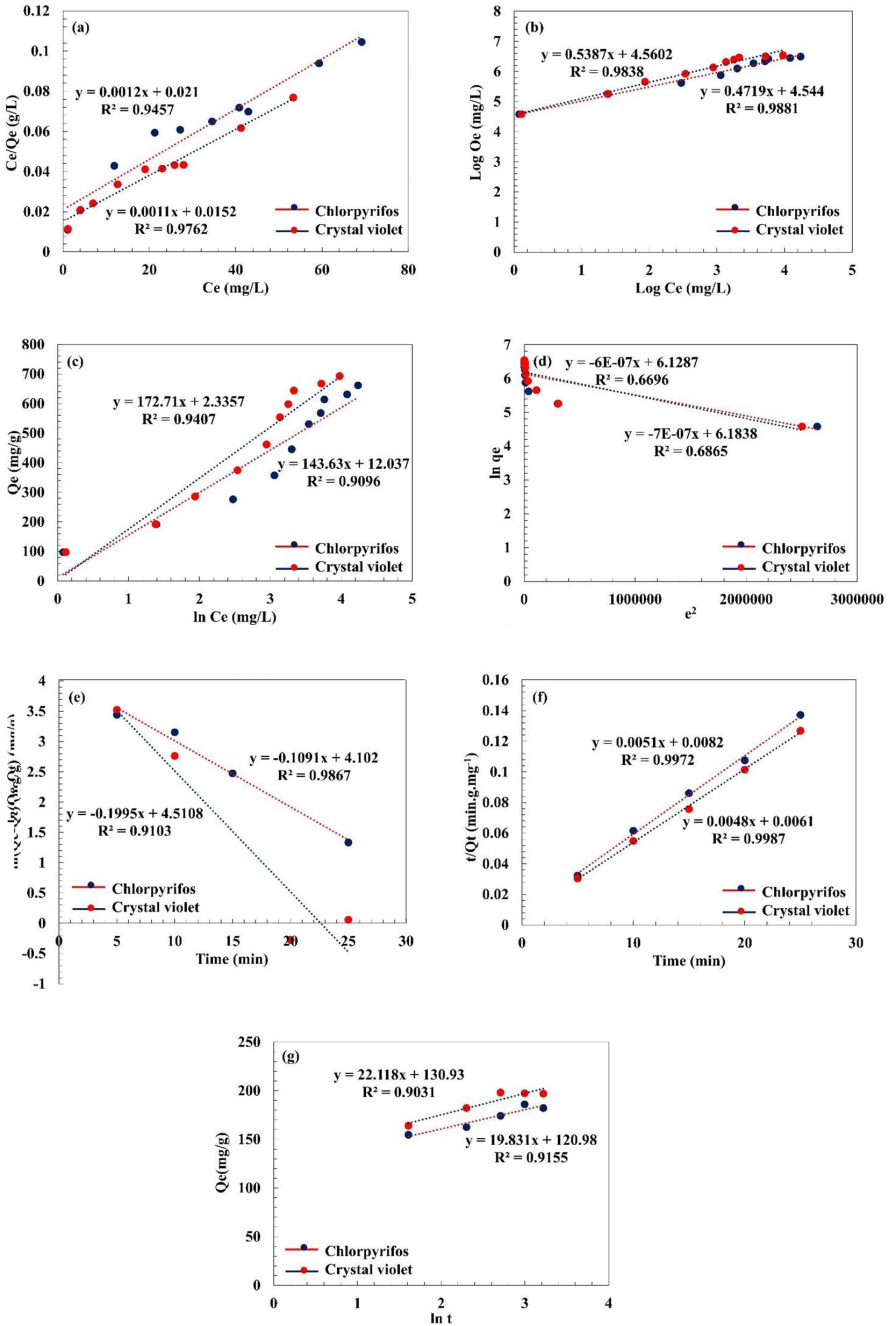
(**a**) Langmuir, (**b**) Freundlich, (**c**) Temkin, and (**d**) Dubinin-Raduskevich (D-R) isotherms (condition: initial concentration (50–400 mg/L), pH 7 and 8, adsorbent dosage (0.005 g), contact time (20 and 15 min), T (298 K)). (**e**) Pseudo-first-order, (**f**) Pseudo-second-order, and (**g**) Elovich models (conditions: contact time (5–25 min), pH 7 and 8, adsorbent dosage (0.005 g), initial concentration (50 mg/L), T (298 K)) for CPF and CV respectively.

The adsorption isotherms were perused to investigate the interactions between the pectin hydrogel@Fe_3_O_4_-bentonite nanoadsorbent and CPF pesticide and CV dye. Langmuir, Freundlich, Temkin, and Dubinin-Radushkevich (D-R)’s isotherms have been investigated to determine equilibrium adsorption isotherms and compute the highest amount of the adsorption capacity^81^. Langmuir isotherm (Eq. (4)) is a model to describe the one-layer contaminant adsorption on the adsorbent’s surface. In this case, all of the adsorbent’s superficial sites have similar energy and affinity to interact effectively with contaminants, leading to homogeneous adsorption^17^. Indeed, the maximum adsorption capacity is considered when a complete monolayer of the contaminants is formed on the adsorbent’s superficial. On the opposite, the Freundlich isotherm (Eq. (5)) defines as a model to describe multilayer adsorption of pollutants on the heterogeneous surface of the adsorbent. Temkin isotherm (Eq. (6)) defines how the adsorbate substances and the adsorbent’s system have interacted and the adsorption procedure’s bonding energies. In this isotherm, the adsorption energy between the adsorbent’s covered surface and adsorbate diminished based on the descending linear pattern. The Temkin model represents the adsorption procedure’s free energy as an adsorbent’s surface coating function^82^. The Dubinin-Radushkevich (D-R) model (Eqs. (7), (8)) is an empirical isotherm utilized to describe the adsorption procedure based on the pore-filling mechanism. The wide applicability of this isotherm is to represent the adsorption mechanism executed onto homogeneous and heterogeneous surfaces^83^. The equations of the Langmuir, Freundlich, Temkin, and Dubinin-Radushkevich (D-R) isotherms are defined in Eqs. (4), (5), (6), (7) and (8) , respectively^58^.

4 $$\frac{{C}_{e}}{{Q}_{e}}=\frac{1}{{K}_{L}{Q}_{max}}+\frac{1}{{Q}_{max}}{C}_{e}$$

5 $$Log{Q}_{e}=Log{K}_{F}+\frac{1}{n}Log{C}_{e}$$

6 $${Q}_{e}= \frac{RT}{{b}_{T}} (Ln{K}_{T}+ Ln{C}_{e})$$

7 $${q}_{e}= {q}_{s}\mathrm{exp}(-{K}_{DR}{\varepsilon }^{2})$$

8 $$\varepsilon =RTln(1+\frac{1}{{C}_{e}})$$

where C_e_ (mg/L) belongs to the equilibrium concentration of CPF and CV, Q_e_ (mg/g) is the equilibrium adsorption capacity, and Q_max_ (mg/g) represents the highest adsorption capacity. K_L_ (L/mg) and K_F_ (L/mg) are constants in Langmuir and Freundlich isotherms computed from the plot between C_e_/Q_e_ and C_e_, and between log Q_e_ and log C_e_, respectively. In the case of n > 1, the CPF and CV’s adsorption at high concentrations on the adsorption surface is favorable^17^. For the Temkin isotherm (Eq. (6)), R represents the universal gas constant, T (K) stands for the temperature, b_T_ is ascribed to the adsorption heat, and K_T_ (L.mg^−1^) is the constant of the Temkin model. In Eqs. (7), (8) for Dubinin-Radushkevich (D-R) isotherm model, q_s_ (mg P/g) is adsorption capacity-related Dubinin-Radushkevich (D-R)’s constant, K_DR_ (mol^2^/kJ^2^) stands for adsorption’s average free energy, R (J/mol K) stands for the gas constant, and T (K) is the temperature. According to the charts of the Langmuir, Freundlich, Temkin, and Dubinin-Radushkevich (D-R) isotherms in Fig. 10a–d, respectively, and based on the data presented in informative Table 3, obtained from Langmuir, Freundlich, Temkin, and Dubinin-Radushkevich (D-R) isotherms, Freundlich isotherm well matches the experimental information compared to Langmuir, Temkin, and Dubinin-Radushkevich (D-R) isotherms for CPF and CV. The adsorption kinetics linear plots and the computed CPF and CV contaminants’ parameters were exhibited in Fig. 10e–g. Regarding the R^2^ term, which is corresponded to the correlation coefficient and various amounts of Q_e,experimental,_ and Q_e,calculated_ for CPF and CV pollutants, pseudo-second-order models described the adsorption kinetics. Besides, the R^2^ term was reported to be 0.9972 and 0.9987 for CPF and CV, respectively, indicating a close value to the unit that matched the pseudo-second-order kinetics model compared to the R^2^ term calculated from the pseudo-second-order (CPF: 0.9867, CV: 0.9103) and Elovich (CPF: 0.9155, CV: 0.9031) model. Moreover, Table 4 compares CPF and CV’s adsorption capacity on the prepared pectin hydrogel@Fe_3_O_4_-bentonite nanoadsorbent with other adsorbents reported in prior studies. Among all of the described adsorbents investigated previously, the prepared pectin hydrogel@Fe_3_O_4_-bentonite nanoadsorbent demonstrated a desirable Q_max_. Various physicochemical characteristics of the prepared nanoadsorbent, such as facile contaminant diffusion into the 3D cross-linked network and mesoporous structure of the magnetic hydrogel-based nanoadsorbent, a large surface area mainly owed to the bentonite addition, numerous adsorption reactive sites, such as hydroxyl and carboxylate groups, and nanosized Fe_3_O_4_ NPs are some of the reasons that cause a great Q_max_ amount. Therefore, the prepared pectin hydrogel@Fe_3_O_4_-bentonite nanoadsorbent is recommended for adsorbing the organophosphorus pesticide and toxic dye pollutants from wastewater.

**Table 3:** Isotherm and kinetic constants, and correlation coefficients for CPF and CV adsorption on the pectin hydrogel@Fe_3_O_4_-bentonite nanoadsorbent.

Model		Parameters	CPF	CV
Isotherm	Freundlich	K_F_, mg.g^−1^	94.0663	95.6026
		n	2.11909	1.85632
		R^2^	0.9881	0.9838
	Langmuir	Q_max_ (mg/g)	833.333	909.091
		K_L_ (L/mg)	0.05714	0.07237
		R^2^	0.9457	0.9762
	Temkin	K_T_ (L/g)	1.087417	1.013616
		b_T_ (J/mol)	143.63	172.71
		R^2^	0.9096	0.9407
	Dubinin-Radushkevich (D-R)	β (mol^2^/K^2^J^2^)	6.1865E-7	6.1835E-7
		E (KJ/mol)	899.0058	855.2446
		Q_m_ (mg/g)	458.8393	484.8405
		R^2^	0.6696	0.6865
Kinetics	Pseudo-first-order	K_1_ (min^−1^)	0.1091	0.1995
		Q_e, experimental_ (mg/g)	185.98	198.04
		Q_e, calculated_ (mg/g)	60.46109	90.46109
		R^2^	0.9867	0.9103
	Pseudo-second-order	k_2_ (min^−1^)	0.003172	0.003777
		Q_e, experimental_ (mg/g)	185.98	198.04
		Q_e, calculated_ (mg/g)	196.0784	208.333
		R^2^	0.9972	0.9987
	Elovich	α	0.193293	41.36307
		β	0.050426	0.045212
		R^2^	0.9155	0.9031

**Table 4:** Evaluation of pectin hydrogel@Fe_3_O_4_-bentonite nanoadsorbent with previous reports.

Adsorbent	Q_max_ (mg/g) CV	Ref	Adsorbent	Q_max_ (mg/g) CPF	Ref
Alginate/bentonite beads	498.20	^84^	TC4As-XG@Fe_3_O_4_	769.23	^58^
Chitosan coated bentonite	169.49	^85^	Arabic gum-g-polyamidoxime/CuFe_2_O_4_	769.23	^6^
Raw bentonite	131.00	^86^	Co_3_O_4_/PANI-6 wt% Sm_2_O_3_	96.73	^87^
M-MoS_2_@bentoniteNC	384.61	^88^	Cloisite 20A	6.63	^89^
Guar gum/bentonite	167.93	^90^	Fe_3_O_4_@SiO_2_@GO-PEA	25.6	^91^
Bentonite–alginate	462.60	^92^	N-Bent-NFe_3_O_4_-Sod.Alg	29.17	^93^
Pec-g-poy(AMPS-co-AAm)/ZnO	568.33	^94^	Polyvinylamine-modified nanocellulose	98.11	^95^
Mag./silica/pectin NPs	125.00	^96^	Magnetic molecularly imprinted polymer	172.41	^97^
Khulays natural bentonite	263.00	^98^	Fe_3_O_4_@SiO_2_@GO-PEA (2-phenylethylamine)	25.6	^99^
Pectin hydrogel@Fe_3_O_4_-bentonite	909.091	Present work	pectin hydrogel@Fe_3_O_4_-bentonite	833.333	Present work

### Recyclability of pectin hydrogel@Fe_3_O_4_-bentonite magnetic mesoporous nanoadsorbent in adsorption process and interfaces assessment

**Figure 11:**
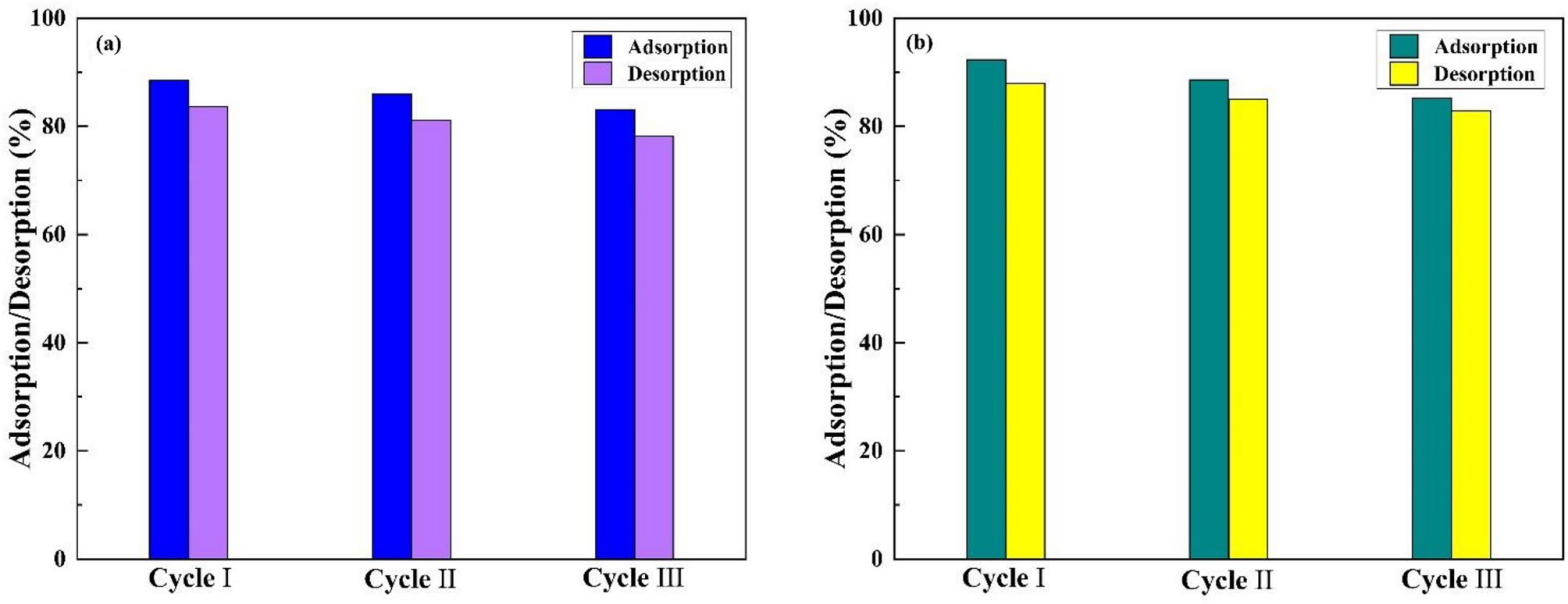
The recyclability diagram of the pectin hydrogel@Fe_3_O_4_-bentonite magnetic nanoadsorbent for eliminating (**a**) CPF and (**b**) CV for three successive runs.

The retrievability of nanoadsorbent is highly significant for application in industrial facets^17^. According to the attained results, as shown in Fig. 11, the recyclability of pectin hydrogel@Fe_3_O_4_-bentonite magnetic mesoporous nanoadsorbent was accomplished for three consecutive adsorption–desorption cycles. To peruse the desorption, the magnetic nanoadsorbents were added into the HCl solution with 0.1 M concentration and shaken for desorption at room temperature separately to dissociate the nanoadsorbent-CV and nanoadsorbent-CPF bonds. Then, the magnetic nanoadsorbents were isolated by a magnet, rinsed with distilled water, and dried in an oven at 60 °C. Due to the consecutive reusability cycles in three runs, no substantial decrease was detected in the CPF and CV adsorption capacity. In this line, the pectin hydrogel@Fe_3_O_4_-bentonite magnetic mesoporous nanoadsorbent is a great system for removing CPF and CV, and it can be conveniently collected without any considerable diminish in adsorption efficiency.

After the CPF and CV adsorption at optimum conditions, the pectin hydrogel@Fe_3_O_4_-bentonite nanoadsorbent was filtered, and the ICP test was taken from the filtrate. Based on the ICP results, the Fe^3+^ and Ca^2+^ ions concentration released into the filtrate solution was measured to be 3.21 ppm and 2.09 ppm, respectively. This meager leaching amount is ascribed to the incomplete magnetic nanoadsorbent separation from the reaction mixture after completing the adsorption with a magnet. Hence, the released ions to the filtrate solution were so few, indicating the nanoadsorbent's stability and applicability to be recycled for three successive cycles without any loss in adsorption efficiency.

### Proposed mechanism of adsorption

**Figure 12:**
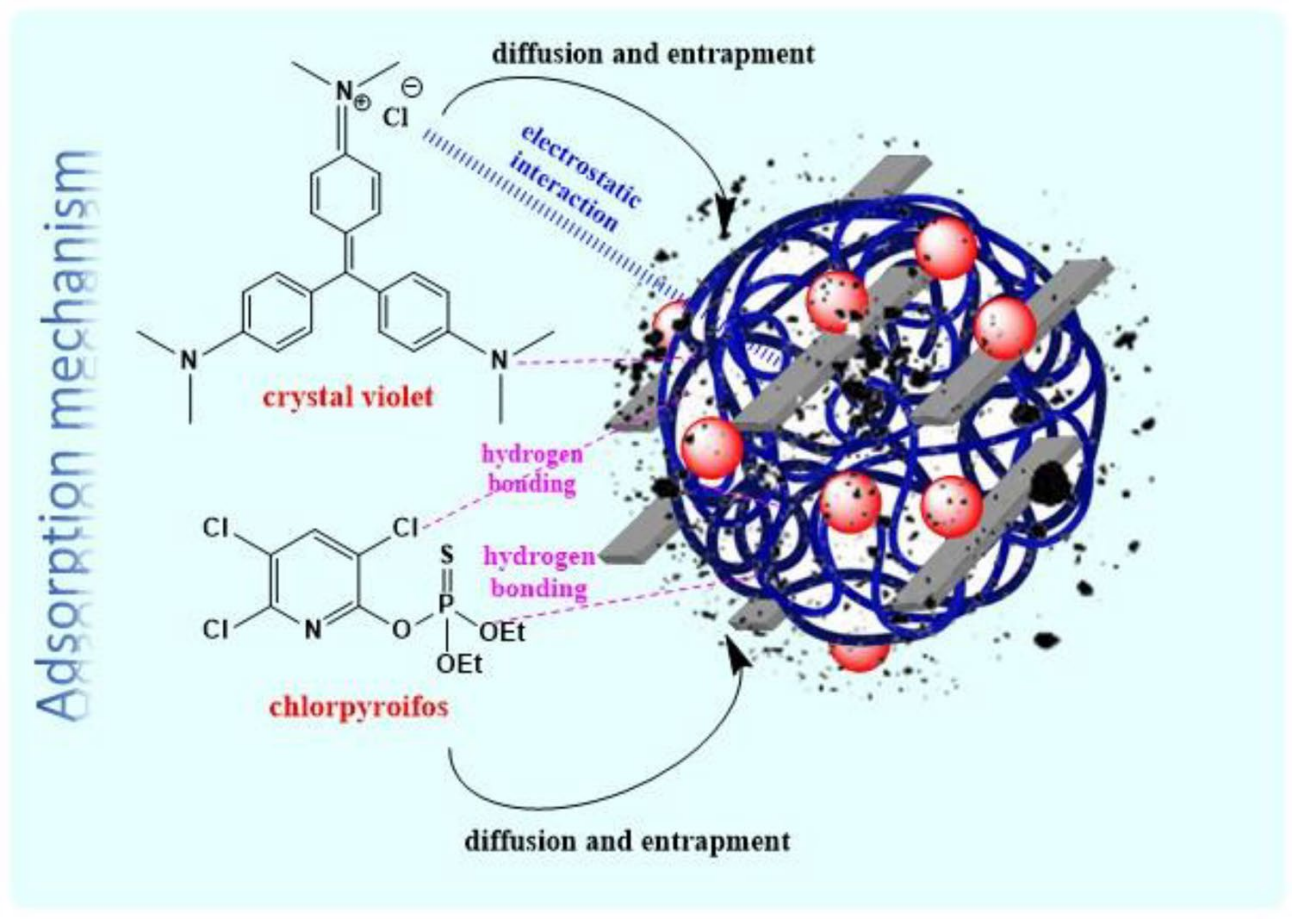
Possible adsorption mechanism of CV and CPF onto the pectin hydrogel@Fe_3_O_4_-bentonite magnetic nanoadsorbent.

As previously stated, the pectin hydrogel@Fe_3_O_4_-bentonite magnetic nanoadsorbent has a prominent role in organic dye and pesticide elimination according to its enhanced surface area and mesoporosity. Additionally, abundant hydroxyl and carboxyl functional groups of the pectin hydrogel tend to provide a hydrogen bond network with CV and CPF. The electrostatic interaction between CPF and the magnetic nanoadsorbent occurs due to their heteroatom structures. It should be considered that based on the anionic nature of the prepared nanoadsorbent in different acidic, neutral, and alkaline solution pH, as shown in Table 2, and the cationic charge of the CV, the leading interaction between CV and the prepared magnetic nanoadsorbet would be an electrostatic attraction (Fig. 12). Notably, the highest adsorption efficiency related to the most effective electrostatic interaction between the nanoadsorbent and CV occurred at pH = 8 because, in the acidic pH, the produced H^+^ in the solution acts as a competing species with positively-charged CV for proper binding sites on the surface of the prepared adsorbent; therefore, in acidic pH, the adsorption efficiency reduces. Furthermore, with a quick glance at the remarkable porous structure of the cross-linked pectin hydrogel, plausible physicochemical adsorption, and BET surface area of the nanoadsorbent (68.904 m^2^/g), which mainly arose from the bentonite addition to the nanocomposite, it can be deduced that CPF and CV can be physically trapped in the nanoadsorbent structure. Overall, numerous pores, OH and COOH groups, and also the heteroatom structure of the nanoadsorbent have promoted the affinity and adsorption capacity toward CPF and CV contaminants.

## Conclusion

In this work, the prepared pectin hydrogel@Fe_3_O_4_-bentonite magnetic nanoadsorbent demonstrated an excellent magnetism, due to the *presence of* the Fe_3_O_4_ MNPs in its structure. The magnetic nanoadsorbent was characterized via different analatical approaches, such as FTIR, EDX, FESEM, XRD, TGA, BET, and VSM to find out the precise structural properties of the prepared nanoadsorbent. The adsorption efficiency of the organophosphorus CPF and CV organic dye in an aqueous medium was calculated. The CPF and CV adsorption capacity was 833.333 mg/g and 909.091 mg/g in optimum conditions, respectively. TGA curve displayed ca. 15% weight loss to 800 °C, indicating the enhanced thermal stability of the pectin hydrogel@Fe_3_O_4_-bentonite nanoadsorbent. Besides, XRD patterns clearly show the immobilization and uniformity in the distribution of Fe_3_O_4_ MNPs in the nanoadsorbents, enhancing the crystallinity of the structure even after adding bentonite. Due to the VSM results, the magnetic saturation of the nanoadsorbent was 20.53 emu/g without magnetic Remanence and coercivity, and it represented the superparamagnetic properties. The FESEM images showed the uniformity in size and shape of the prepared Fe_3_O_4_ MNPs throughout the cross-linked structure of pectin hydrogel. Also, after bentonite addition, smoothing the pore’s surface and the formation of the layered structure in FESEM images are evident. The pectin hydrogel@Fe_3_O_4_-bentonite nanoadsorbent was introduced as an effective adsorption system for eliminating CPF and CV from aqueous media based on the enhanced surface area, numerous active interaction sites, and effective interactions with contaminants. Generally, the prepared nanoadsorbent was an appropriate material to scale up and industrialization regarding the convenient isolation from the reaction, preparation from affordable substances, efficient binding to the pollutants, and maintenance the structural stability after three consecutive retrievability cycles with mo remarkable decrease in the adsorption yield.

## Supplementary Information

Supplementary Information.

## Data Availability

All data generated or analysed during this study are included in this submitted article (and its Supplementary Information files).
