# Supplementary material for: Enhancement of adsorption efficiency of crystal violet and chlorpyrifos onto pectin hydrogel@Fe3O4-bentonite as a versatile nanoadsorbent
Source: Sci Rep. 2023 Jul 4;13:10764. doi: 10.1038/s41598-023-38005-z (PMC10319861; doi:10.1038/s41598-023-38005-z)
Supplement: Supplementary file 1 — Supplementary Information. [file 41598_2023_38005_MOESM1_ESM.docx]

**Supporting information**

**Enhancement of adsorption efficiency of Crystal violet and Chlorpyrifos onto Pectin hydrogel@Fe_3_O_4_-bentonite as a versatile nanoscale adsorbent**

*Paria Beigi^a^,* *Fatemeh Ganjali^b^,* *Fereshte Hassanzadeh-Afruzi^b,1^,* *Mohammad Mehdi Salehi^b^, Ali Maleki^b,*^*

*^a^Department of Physics, Iran University of Science and Technology, Tehran 16846–13114, Iran*

*^b^Catalysts and Organic Synthesis Research Laboratory, Department of Chemistry, Iran University of Science and Technology, Tehran 16846-13114, Iran.*

*^1^Co-first author*

**Corresponding author: E-mail: maleki@iust.ac.ir; Fax: +98-21-73021584; Tel: +98-21-73228313.*

*
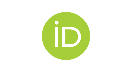
Author’s ORCIDs:*

*Fereshteh Hasanzadeh-Afruzi:* [*https://orcid.org/0000-0003-0570-2506*](https://orcid.org/0000-0003-0570-2506)

*Fatemeh Ganjali: https://orcid.org/0000-0002-2594-2761*

*Mohammad Mehdi Salehi: https://orcid.org/0000-0003-3648-1865*

*Ali Maleki:* [*https://orcid.org/0000-0001-5490-3350*](https://orcid.org/0000-0001-5490-3350)

| **Content** | **Page** |
| --- | --- |
| **Figure S1.** The calibration curve of organophosphorus CPF pesticide. | **S2** |
| **Figure S2:** The calibration curve of organic CV dye. | **S3** |
| **Figure S3:** FT-IR spectra. | **S4** |
| **Figure S4:** EDX analysis. | **S5** |
| **Figure S5:** XRD pattern. | **S6** |
| **Figure S6.** FESEM image. | **S7** |
| **Figure S7.** magnetization curve. | **S8** |
| **Figure S8.** TGA curves. | **S9** |
| **Figure S9.** BET curves. | **S10** |
| **Figure S10.** Effective factors on the CPF and CV adsorption by pectin hydrogel@Fe_3_O_4_-bentonite magnetic nanoadsorbent. | **S11** |
| **Figure S11.** Adsorption isotherm and adsorption kinetics studies. | **S12** |
| **Figure S12.** The recyclability diagram of the pectin hydrogel@Fe_3_O_4_-bentonite magnetic nanoadsorbent for eliminating a) CV and b) CPF for three successive runs in terms of adsorption capacity. | **S13** |
| **Figure S13.** The recyclability diagram of the pectin hydrogel@Fe_3_O_4_-bentonite magnetic nanoadsorbent for eliminating a) CPF and b) CV for three successive runs. | **S14** |
| **Table S1.** Zeta potential of the pectin hydrogel@Fe_3_O_4_-bentonite nanoadsorbent at various pH and solutions. | **S15** |
| **Table S2.** Isotherm and kinetic constants, and correlation coefficients for CPF and CV adsorption on the pectin hydrogel@Fe_3_O_4_-bentonite nanoadsorbent. | **S16** |
| **Table S3.** Evaluation of pectin hydrogel@Fe_3_O_4_-bentonite nanoadsorbent with previous reports. | **S18** |

**Figure S1:** The calibration curve of organophosphorus CPF pesticide.

**Figure S2:** The calibration curve of organic CV dye.


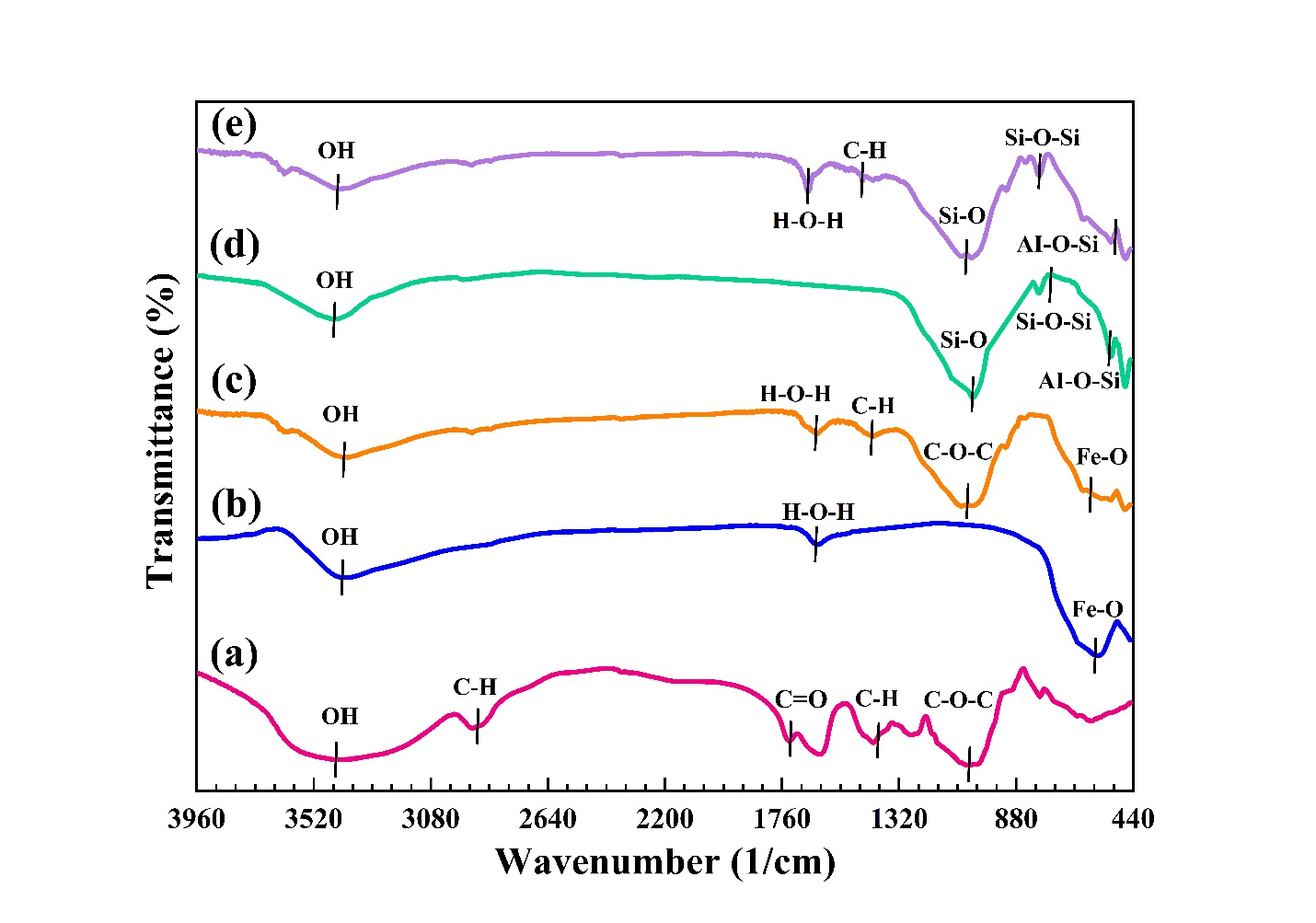


**Figure S3:** The FT-IR spectra of (a) Pectin hydrogel, (b) Fe_3_O_4_ MNPs, (c) Pectin hydrogel@Fe_3_O_4_, (d) bentonite (e) Pectin hydrogel@Fe_3_O_4_-bentonite.

*
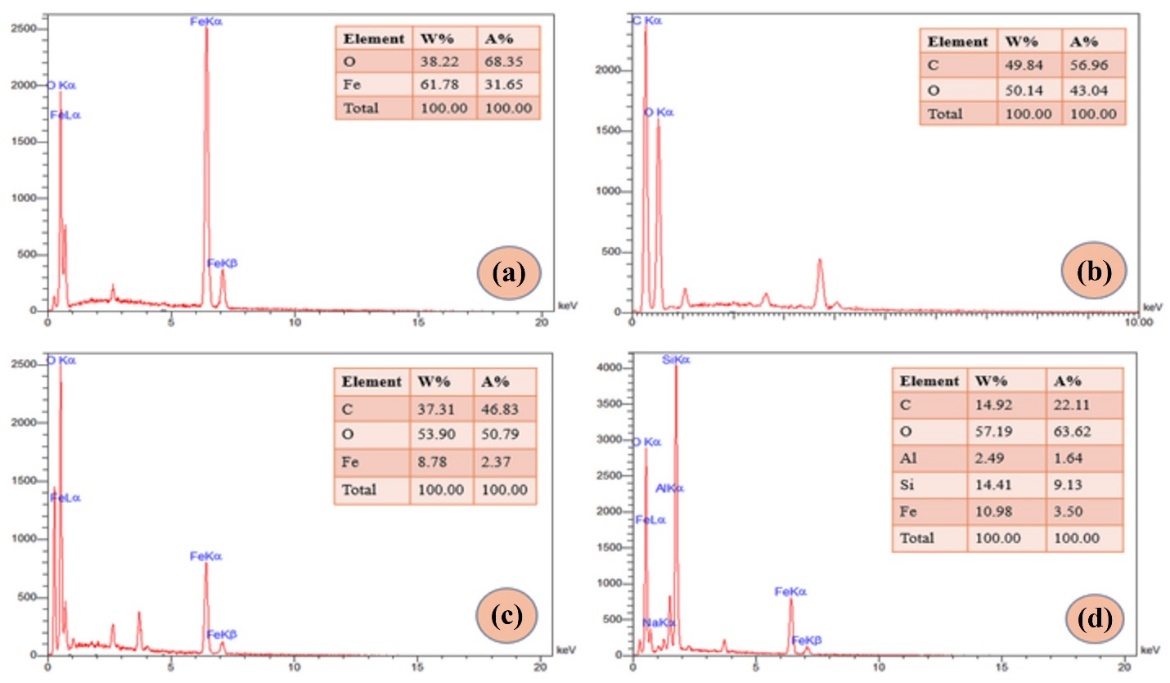
*


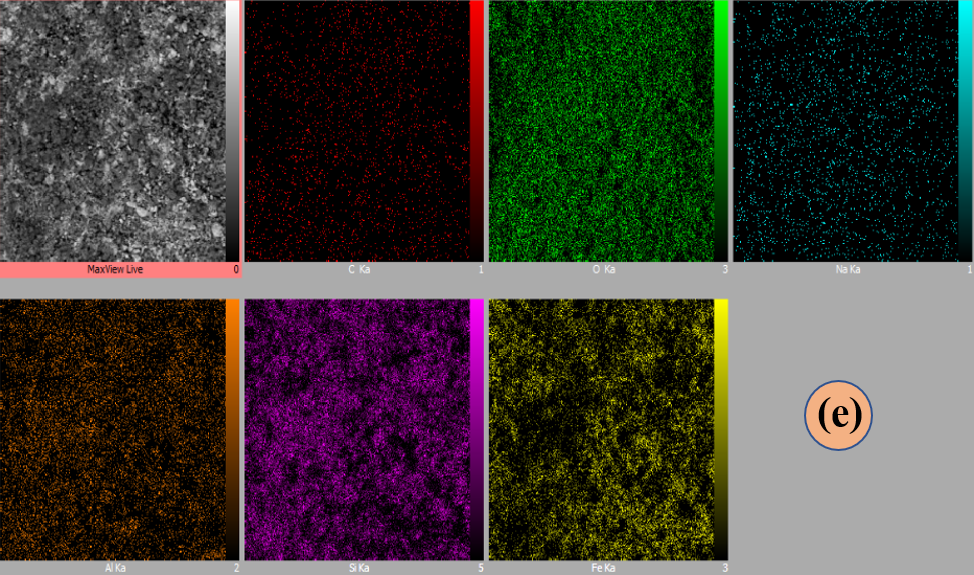


**Figure S4:** EDX analysis of (a) Fe_3_O_4_ MNPs, (b) Pectin hydrogel, (c) Pectin hydrogel@Fe_3_O_4_, (d) Pectin hydrogel@Fe_3_O_4_-bentonite nanoadsorbent, and (e) EDX mapping of Pectin hydrogel@Fe_3_O_4_-bentonite nanoadsorbent.


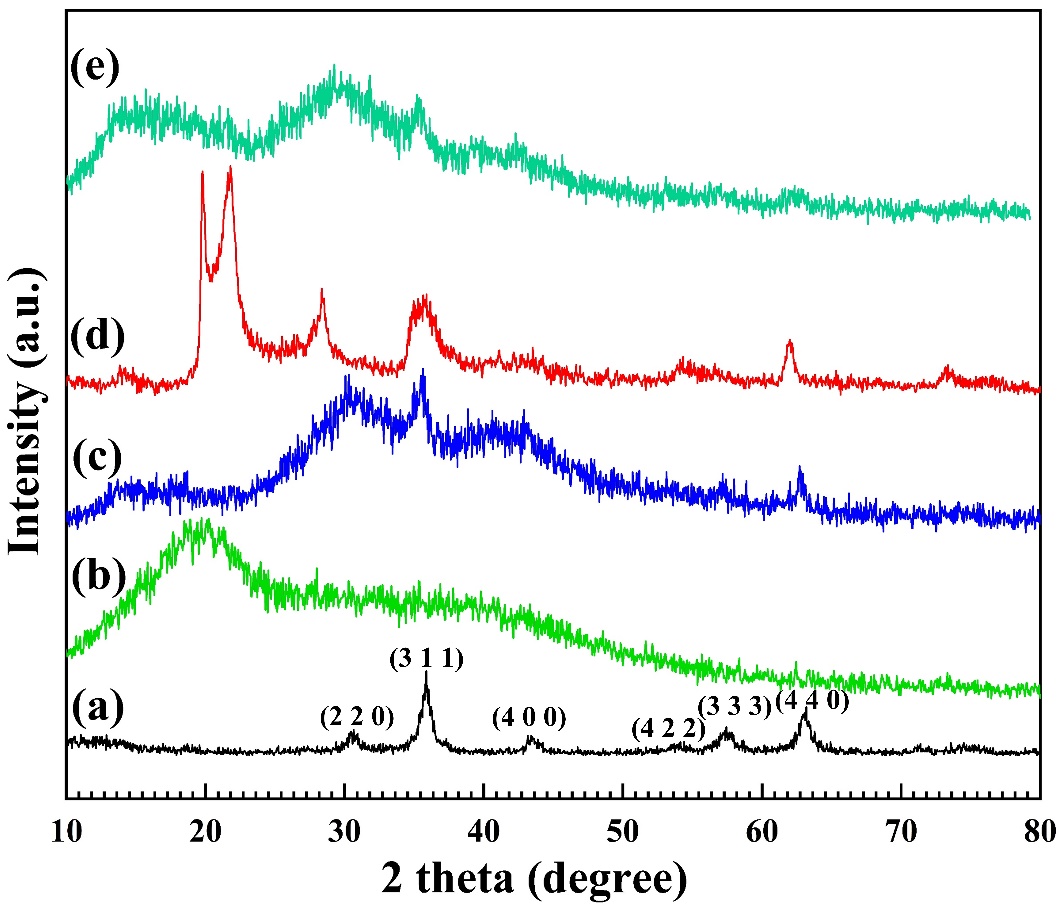


**Figure S5:** XRD pattern of (a) Fe_3_O_4_ MNPs, (b) Pectin, (c) Pectin hydrogel@Fe_3_O_4_, (d) bentonite, (e) Pectin hydrogel@Fe_3_O_4_-bentonite nanoadsorbent.


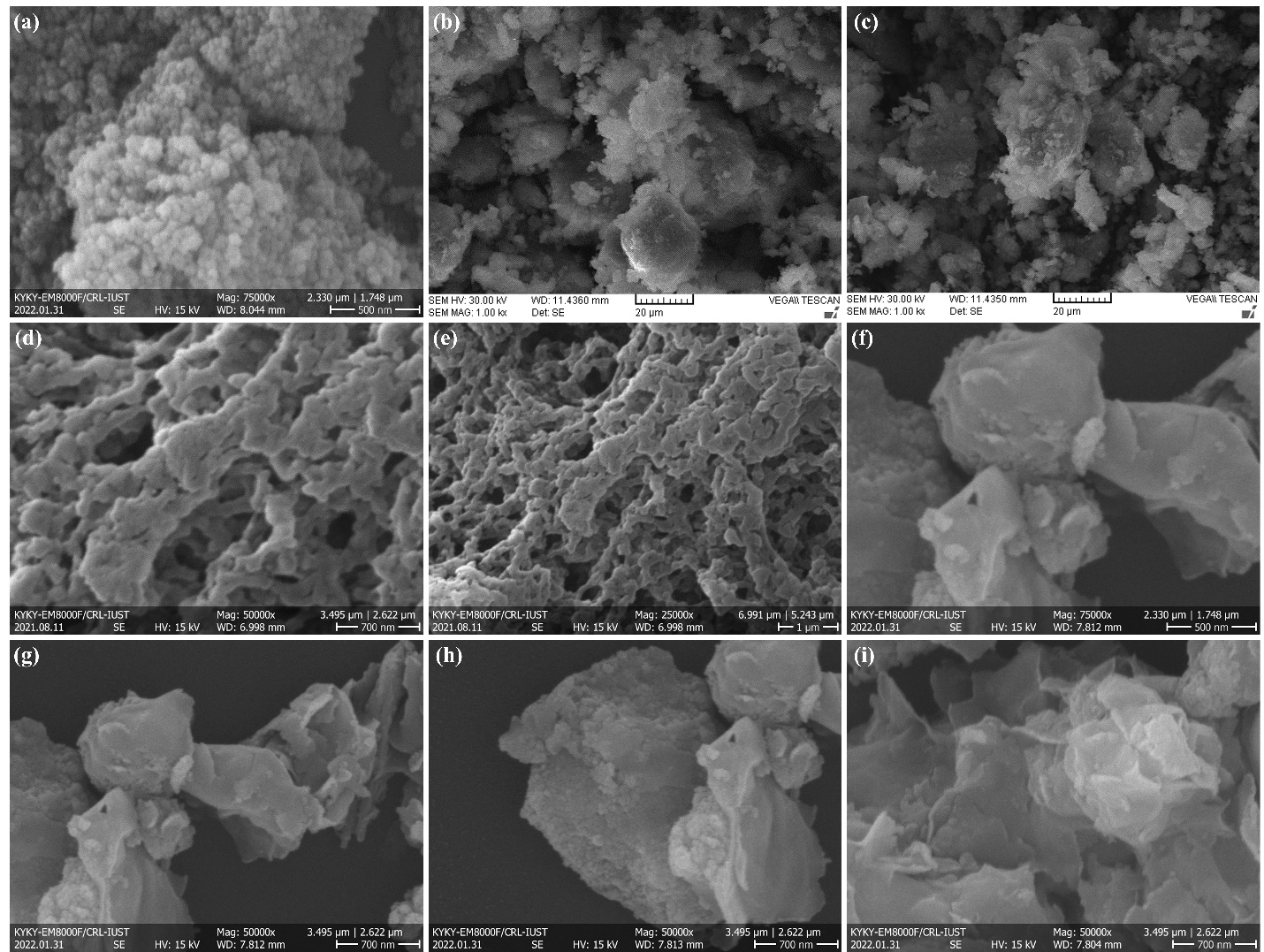


**Figure S6:** FESEM images of (a) Fe_3_O_4_ magnetic nanoparticles, (b, c) Bentonite, (d, e) Pectin hydrogel@Fe_3_O_4_, (f-i) Pectin hydrogel@Fe_3_O_4_-bentonite nanoadsorbent.


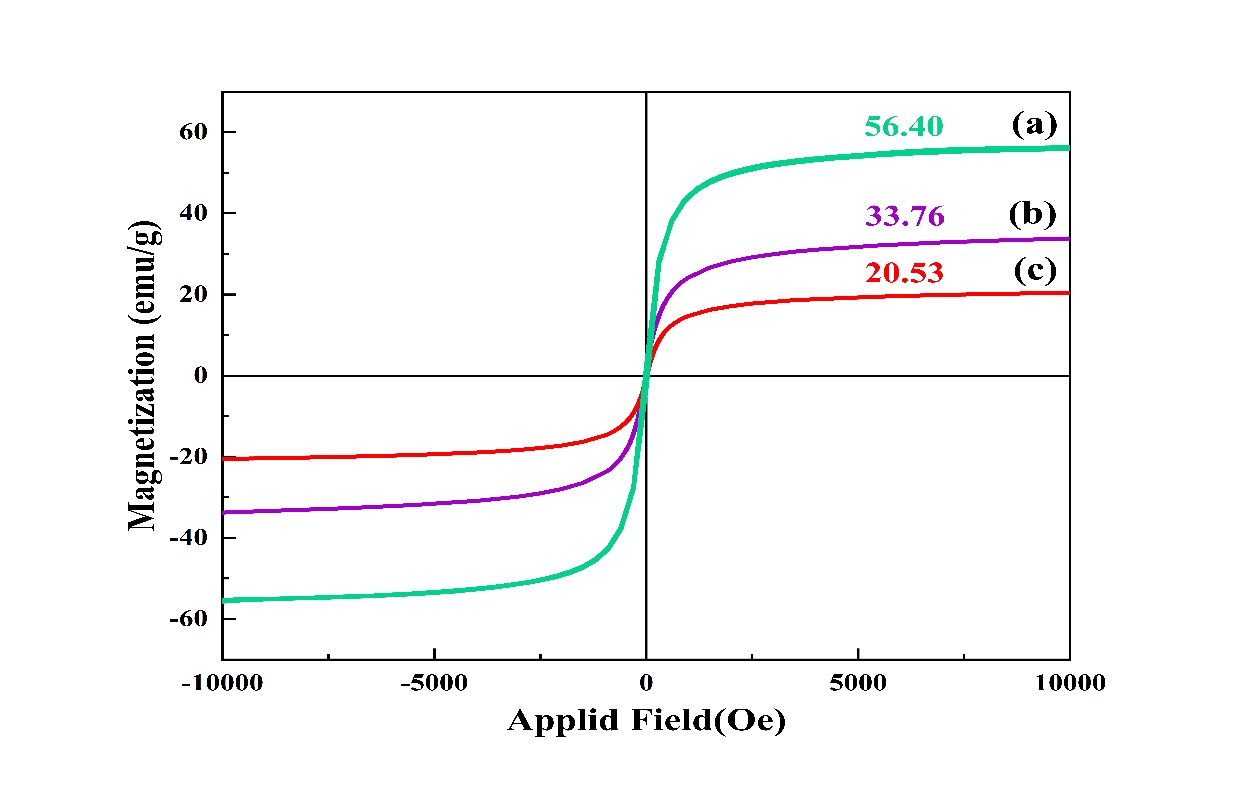


**Figure S7:** Magnetic hysteresis loops of (a) Fe_3_O_4_ MNPs, (b) Pectin hydrogel@Fe_3_O_4_, (c) Pectin hydrogel@Fe_3_O_4_-bentonite nanoadsorbent.


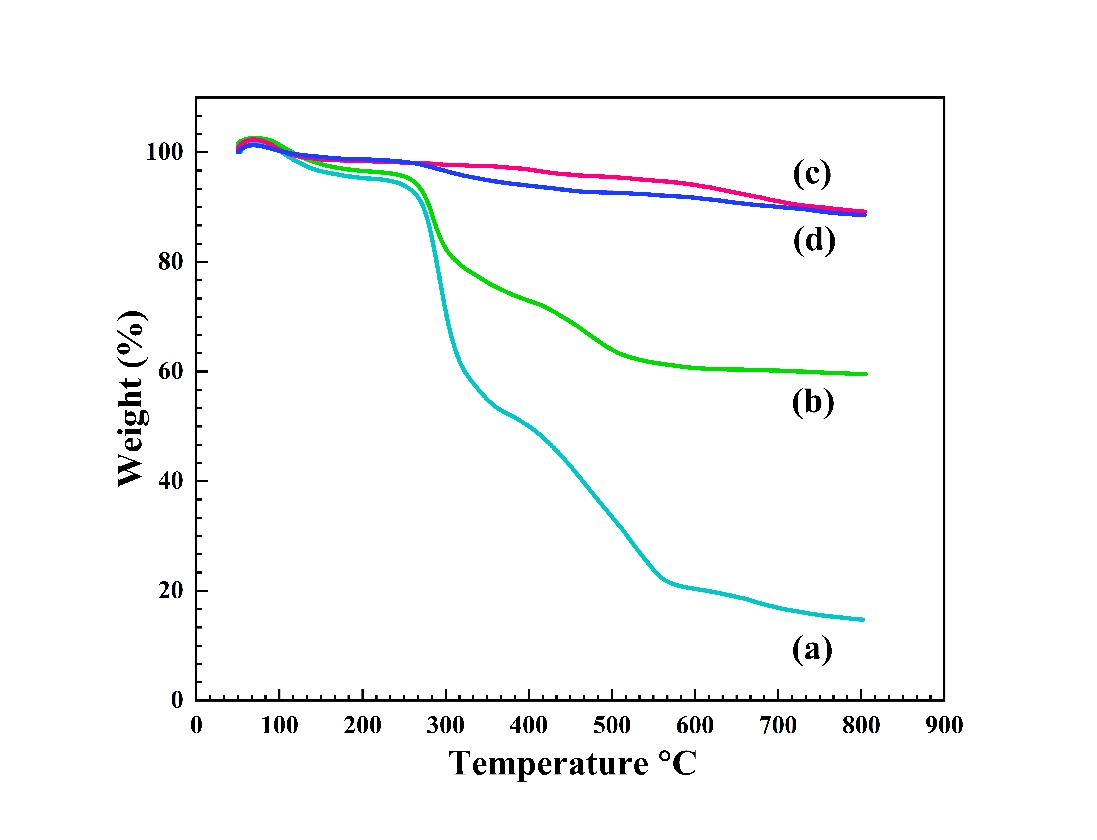


**Figure S8:** TGA curve of (a) pectin hydrogel, (b) Pectin hydrogel@Fe_3_O_4_, (c) bentonite, (d) Pectin hydrogel@Fe_3_O_4_-bentonite nanoadsorbent.


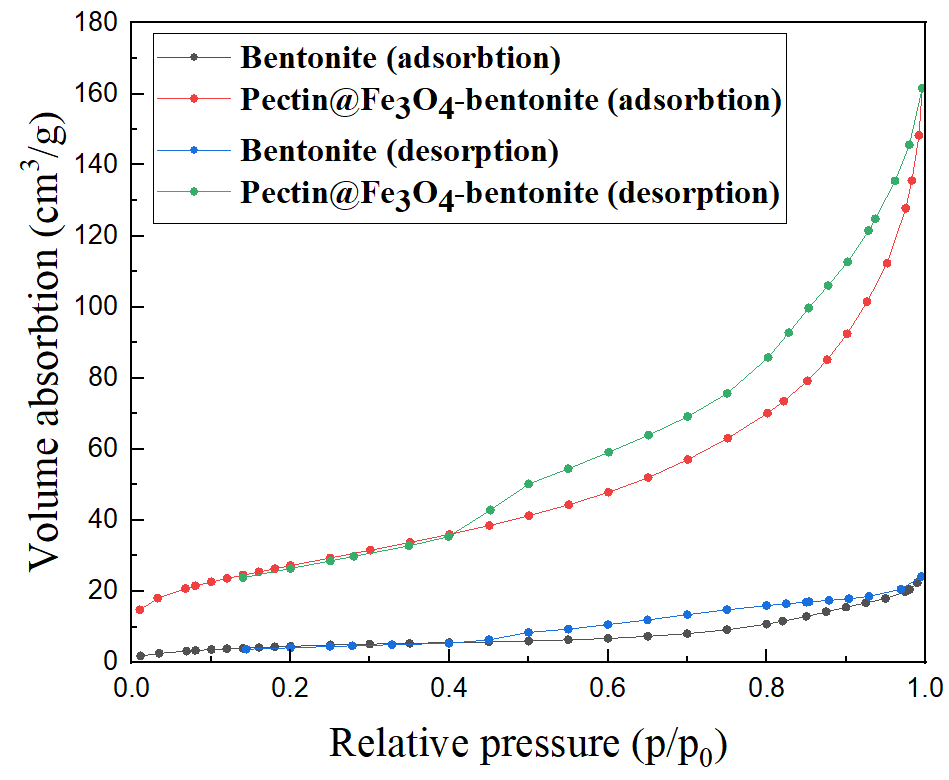


**Figure S9:** The N_2_ adsorption-desorption isotherms of bentonite and pectin hydrogel@Fe_3_O_4_-bentonite nanoadsorbent.

|  |  |
| --- | --- |
|  |  |

| **(a)** | **(b)** |
| --- | --- |
| **(c)** | **(d)** |

**(b)**

**(c)**

**(d)**

**(a)**

**Chlorpyrifos**

**Crystal violet**

**(b)**

**Chlorpyrifos**

**Crystal violet**

**(c)**

**(d)**

**Chlorpyrifos**

**Crystal violet**

**Chlorpyrifos**

**Crystal violet**

**Figure S10:** (a) Effect of solution pH (4–9), adsorbent dosage (0.01 g), initial concentration (100 mg/L), V (10 mL), Time (15 min), T (298 K), (b) adsorbent dosage (0.005–0.025 g), pH 7 and 8 for CPF and CV respectively, initial concentration (100 mg/L), V (10 mL), Time (15 min), T (298 K), (c) contact time (5–25 min), pH 7 and 8 for CPF and CV respectively, adsorbent dosage (0.005 g), initial concentration (100 mg/L), V (10 mL), T (298 K), (d) initial concentration (50–400 mg/L), pH 7 and 8 for CPF and CV respectively, adsorbent dosage (0.005 g), V (10 mL), Time (20 and 15 min for CPF and CV respectively), T (298 K).


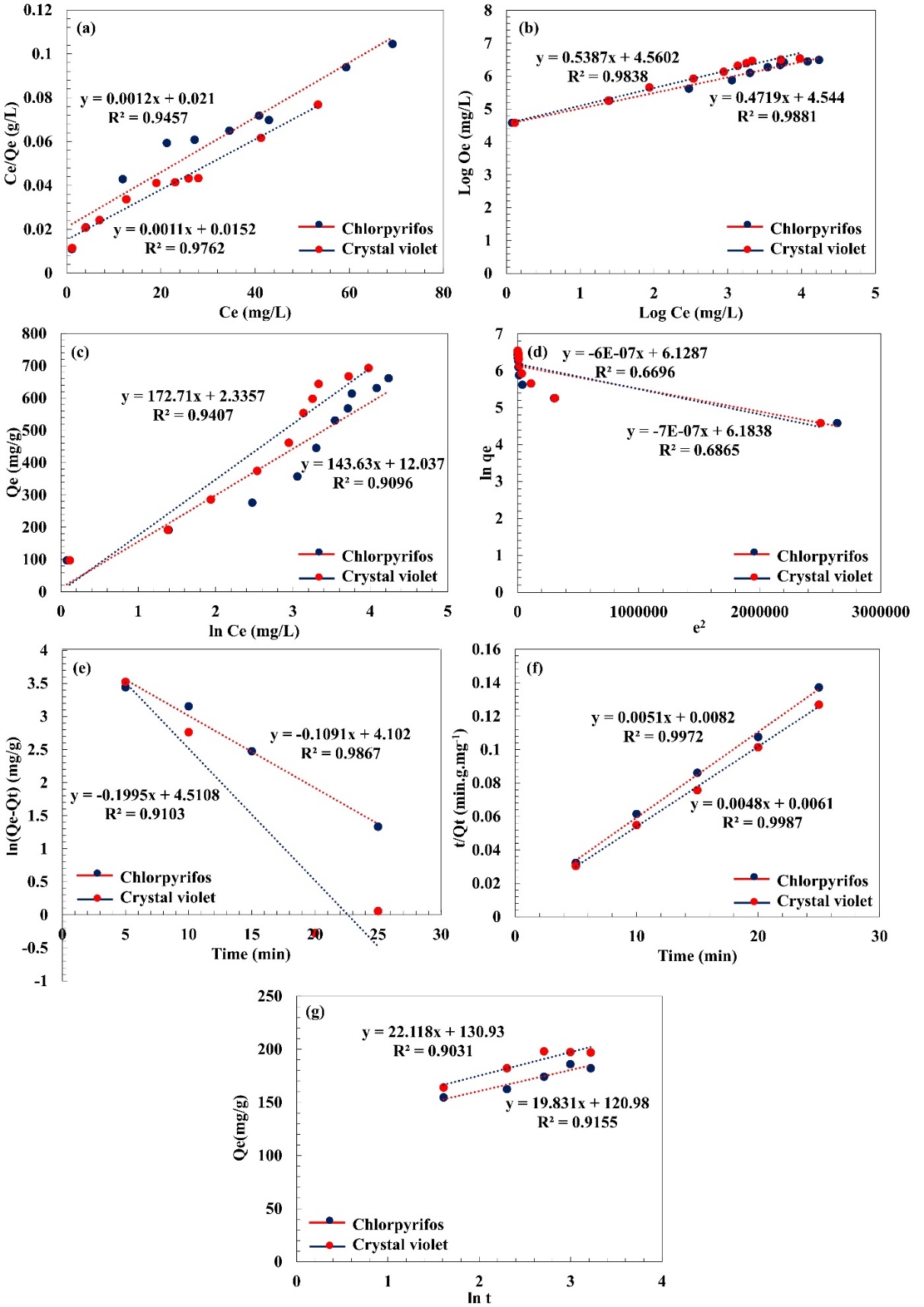


**Figure S11:** (a) Langmuir, (b) Freundlich, (c) Temkin, and (d) Dubinin-Raduskevich (D-R) isotherms (condition: initial concentration (50–400 mg/L), pH 7 and 8, adsorbent dosage (0.005 g), contact time (20 and 15 min), T (298 K)). (e) Pseudo-first-order, (f) Pseudo-second-order, and (g) Elovich models (conditions: contact time (5–25 min), Ph 7 and 8, adsorbent dosage (0.005 g), initial concentration (50 mg/L), T (298 K)) for CPF and CV respectively.


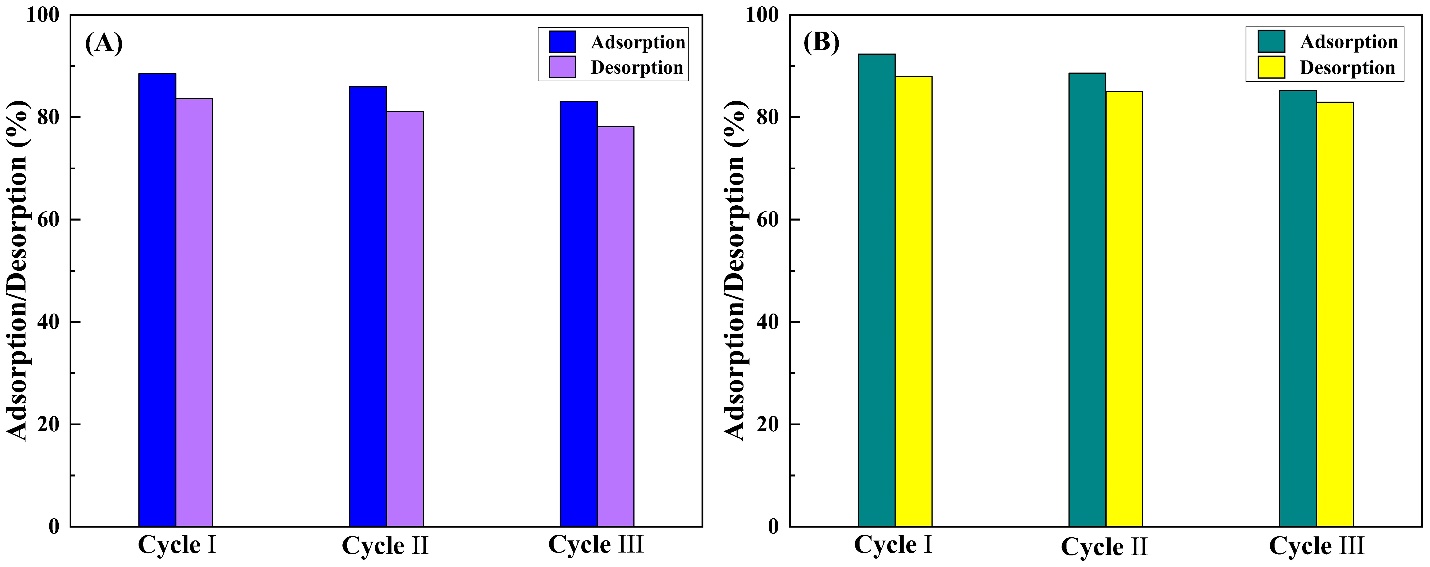


**Figure S12:** The recyclability diagram of the pectin hydrogel@Fe_3_O_4_-bentonite magnetic nanoadsorbent for eliminating a) CPF and b) CV for three successive runs.

**
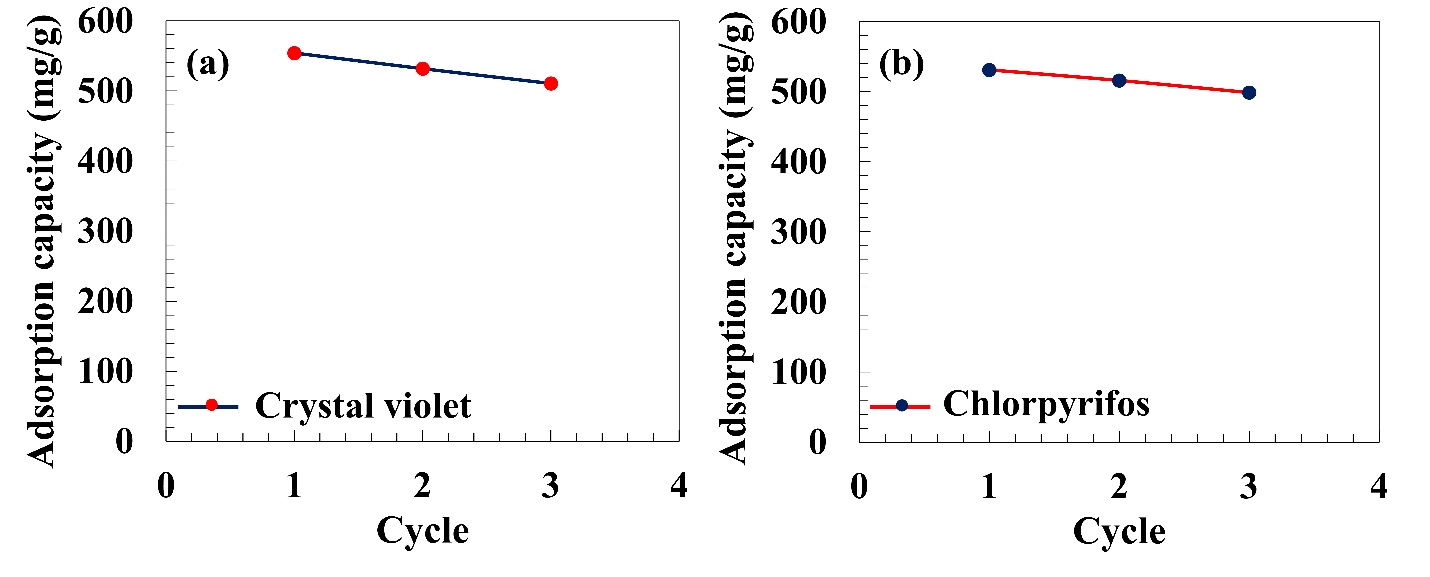
**

**Figure S13.** The recyclability diagram of the pectin hydrogel@Fe_3_O_4_-bentonite magnetic nanoadsorbent for eliminating a) CPF and b) CV for three successive runs.

**Table S1:** Zeta potential of the pectin hydrogel@Fe_3_O_4_-bentonite nanoadsorbent at various pH and solutions.

| **Sample** | **Solution** | **pH** | **Zeta protentional (mV)^a^** |
| --- | --- | --- | --- |
| **Pectin@Fe_3_O_4_-bentonite** | DI water | 3 | -32.2 |
| **Pectin@Fe_3_O_4_-bentonite** | DI water | 5 | -33.1 |
| **Pectin@Fe_3_O_4_-bentonite** | DI water | 7 | -34.1 |
| **Pectin@Fe_3_O_4_-bentonite** | DI water | 9 | -42.8 |
| **Pectin@Fe_3_O_4_-bentonite** | CV | 3 | -32.8 |
| **Pectin@Fe_3_O_4_-bentonite** | CV | 5 | -34.3 |
| **Pectin@Fe_3_O_4_-bentonite** | CV | 7 | -35.9 |
| **Pectin@Fe_3_O_4_-bentonite** | CV | 9 | -36.2 |

^a^The experiment was carried out at room temperature.

**Table S2:** Isotherm and kinetic constants, and correlation coefficients for CPF and CV adsorption on the pectin hydrogel@Fe_3_O_4_-bentonite nanoadsorbent.

| **Model** | | **Parameters** | **CPF** | **CV** |
| --- | --- | --- | --- | --- |
| Isotherm | Freundlich | K_F_, mg.g^-1^ | 94.0663 | 95.6026 |
|  |  | n | 2.11909 | 1.85632 |
|  |  | R^2^ | 0.9881 | 0.9838 |
|  | Langmuir | Q_max_ (mg/g) | 833.333 | 909.091 |
|  |  | K_L_ (L/mg) | 0.05714 | 0.07237 |
|  |  | R^2^ | 0.9457 | 0.9762 |
|  | Temkin | K_T_ (L/g) | 1.087417 | 1.013616 |
|  |  | b_T_ (J/mol) | 143.63 | 172.71 |
|  |  | R^2^ | 0.9096 | 0.9407 |
|  | Dubinin-Radushkevich (D-R) | β (mol^2^/K^2^J^2^) | 6.1865E-7 | 6.1835E-7 |
|  |  | E (KJ/mol) | 899.0058 | 855.2446 |
|  |  | Q_m_ (mg/g) | 458.8393 | 484.8405 |
|  |  | R^2^ | 0.6696 | 0.6865 |
| Kinetics | Pseudo-first-order | K_1_ (min^-1^) | 0.1091 | 0.1995 |
|  |  | Q_e, experimental_ (mg/g) | 185.98 | 198.04 |
|  |  | Q_e, calculated_ (mg/g) | 60.46109 | 90.46109 |
|  |  | R^2^ | 0.9867 | 0.9103 |
|  | Pseudo-second-order | k_2_ (min^-1^) | 0.003172 | 0.003777 |
|  |  | Q_e, experimental_ (mg/g) | 185.98 | 198.04 |
|  |  | Q_e, calculated_ (mg/g) | 196.0784 | 208.333 |
|  |  | R^2^ | 0.9972 | 0.9987 |
|  | Elovich | α | 0.193293 | 41.36307 |
|  |  | β | 0.050426 | 0.045212 |
|  |  | R^2^ | 0.9155 | 0.9031 |

**Table S3:** Evaluation of pectin hydrogel@Fe_3_O_4_-bentonite nanoadsorbent with previous reports.

| **Adsorbent** | **Q_max_ (mg/g) CV** | **Ref.** | **Adsorbent** | **Q_max_ (mg/g) CPF** | **Ref.** |
| --- | --- | --- | --- | --- | --- |
| Alginate/bentonite beads | 498.20 | ^1^ | TC4As-  XG@Fe_3_O_4_ | 769.23 | ^2^ |
| Chitosan Coated Bentonite | 169.49 | ^3^ | Arabic gum-g-polyamidoxime/CuFe_2_O_4_ | 769.23 | ^4^ |
| Raw bentonite | 131.00 | ^5^ | Co_3_O_4_/PANI-6 wt% Sm_2_O_3_ | 96.73 | ^6^ |
| M-MoS_2_@bentoniteNC | 384.61 | ^7^ | Cloisite 20A | 6.63 | ^8^ |
| Guar gum/bentonite | 167.93 | ^9^ | Fe_3_O_4_@SiO_2_@GO-PEA | 25.6 | ^10^ |
| Bentonite–alginate | 462.60 | ^11^ | N-Bent-NFe_3_O_4_-Sod.Alg | 29.17 | ^12^ |
| Pec-g-poy(AMPS-co-AAm)/ZnO | 568.33 | ^13^ | Polyvinylamine-modified nanocellulose | 98.11 | ^14^ |
| Mag./silica/pectin NPs | 125.00 | ^15^ | Magnetic molecularly imprinted polymer | 172.41 | ^16^ |
| Khulays Natural Bentonite | 263.00 | ^17^ | Fe_3_O_4_@SiO_2_@G  O-PEA (2-  phenylethylamin  e) | 25.6 | ^18^ |
| Pectin hydrogel@Fe_3_O_4_-bentonite | 909.091 | Present work | pectin hydrogel@Fe_3_O_4_-bentonite | 833.333 | Present work |

**References**

1. Oladipo, A. A. & Gazi, M. Enhanced removal of crystal violet by low cost alginate/acid activated bentonite composite beads: Optimization and modelling using non-linear regression technique. *Journal of Water Process Engineering* **2**, 43–52 (2014).

2. Hassanzadeh-Afruzi, F., Ranjbar, G., Salehi, M. M., Esmailzadeh, F. & Maleki, A. Thiacalix[4]arene-functionalized magnetic xanthan gum (TC4As-XG@Fe3O4) as a hydrogel adsorbent for removal of dye and pesticide from water medium. *Sep Purif Technol* **306**, 122700 (2023).

3. Vithalkar, S. H. & Jugade, R. M. Adsorptive removal of crystal violet from aqueous solution by cross-linked chitosan coated bentonite. *Mater Today Proc* **29**, 1025–1032 (2020).

4. Hassanzadeh-Afruzi, F., Maleki, A. & Zare, E. N. Efficient remediation of chlorpyrifos pesticide from contaminated water by superparamagnetic adsorbent based on Arabic gum-grafted-polyamidoxime. *Int J Biol Macromol* **203**, 445–456 (2022).

5. Eren, E. Investigation of a basic dye removal from aqueous solution onto chemically modified Unye bentonite. *J Hazard Mater* **166**, 88–93 (2009).

6. Wassel, A. R., Abdelhameed, R. M., Nasralla, N. H. S. & Abomostafa, H. Impact of Sm2O3 onthe Morphological, Optical, Magnetic, and Pesticide Adsorption of Co3O4/PANI Hybrid Nanocomposites. *ECS Journal of Solid State Science and Technology* **11**, 083009 (2022).

7. Uddin, M. K., Mashkoor, F., AlArifi, I. M. & Nasar, A. Simple one-step synthesis process of novel MoS2@bentonite magnetic nanocomposite for efficient adsorption of crystal violet from aqueous solution. *Mater Res Bull* **139**, 111279 (2021).

8. Suciu, N. A. & Capri, E. Adsorption of chlorpyrifos, penconazole and metalaxyl from aqueous solution by modified clays. *http://dx.doi.org/10.1080/03601230902997543* **44**, 525–532 (2009).

9. Ahmad, R. & Mirza, A. Synthesis of Guar gum/bentonite a novel bionanocomposite: Isotherms, kinetics and thermodynamic studies for the removal of Pb (II) and crystal violet dye. *J Mol Liq* **249**, 805–814 (2018).

10. Wanjeri, V. W. O., Sheppard, C. J., Prinsloo, A. R. E., Ngila, J. C. & Ndungu, P. G. Isotherm and kinetic investigations on the adsorption of organophosphorus pesticides on graphene oxide based silica coated magnetic nanoparticles functionalized with 2-phenylethylamine. *J Environ Chem Eng* **6**, 1333–1346 (2018).

11. Fabryanty, R. *et al.* Removal of crystal violet dye by adsorption using bentonite – alginate composite. *J Environ Chem Eng* **5**, 5677–5687 (2017).

12. El-Sharkawy, R. M., Allam, E. A., Ali, A. S. M. & Mahmoud, M. E. Adsorption study of bisphenol-A and chlorpyrifos onto nanobentonite intercalated with magnetite and sodium alginate: kinetics and isotherm models. *International Journal of Environmental Science and Technology* **19**, 9827–9842 (2022).

13. Kodoth, A. K. & Badalamoole, V. Pectin Based Graft Copolymer–ZnO Hybrid Nanocomposite for the Adsorptive Removal of Crystal Violet. *J Polym Environ* **27**, 2040–2053 (2019).

14. Yang, J. *et al.* Optimization of polyvinylamine-modified nanocellulose for chlorpyrifos adsorption by central composite design. *Carbohydr Polym* **245**, 116542 (2020).

15. Attallah, O. A., Al-Ghobashy, M. A., Nebsen, M. & Salem, M. Y. Removal of cationic and anionic dyes from aqueous solution with magnetite/pectin and magnetite/silica/pectin hybrid nanocomposites: kinetic, isotherm and mechanism analysis. *RSC Adv* **6**, 11461–11480 (2016).

16. Masoumi, A., Hemmati, K. & Ghaemy, M. Recognition and selective adsorption of pesticides by superparamagnetic molecularly imprinted polymer nanospheres. *RSC Adv* **6**, 49401–49410 (2016).

17. Al-Shahrani, S. Phenomena of Removal of Crystal Violet from Wastewater Using Khulays Natural Bentonite. *J Chem* **2020**, (2020).

18. Wanjeri, V. & Wayayi, O. NANOCARBON ANCHORED ON MAGNETIC IRON OXIDE-SILICA CORE SHELL NANOCOMPOSITE FOR EXTRACTION OF ORGANOPHOSPHATE PESTICIDES FROM NATURAL WATER.
